# Flaw Sensitivity of Tough and Self-Healing Hydrogels with Hierarchical Structure

**DOI:** 10.1021/polymscitech.5c00018

**Published:** 2025-04-23

**Authors:** Reina Watanabe, Haruna Tsuchibora, Ryuji Kiyama, Kunpeng Cui, Xueyu Li

**Affiliations:** † Laboratory of Soft & Wet Matter, Division of Soft Matter, Graduate School of Life Science, 12810 Hokkaido University, Sapporo 060-0810, Japan; ‡ Laboratory of Soft & Wet Matter, Faculty of Advanced Life Science, 12810 Hokkaido University, Sapporo 001-0021, Japan; § Laboratoire de Sciences et Ingénierie de la Matière Molle, CNRS, ESPCI Paris, PSL Research University, 10 rue Vauquelin, 75005 Paris, France; ∥ Department of Polymer Science and Engineering, 310301 University of Science and Technology of China, Hefei 230026, China; ⊥ Institute for Chemical Reaction Design and Discovery (WPI-ICReDD), 12810 Hokkaido University, Sapporo 001-0021, Japan; # Hefei National Research Center for Physical Sciences at the Microscale, 310301 University of Science and Technology of China, Hefei 230026, China

**Keywords:** viscoelastic, hierarchical structure, flaw sensitivity, crack blunting, birefringence

## Abstract

Unintentional flaws in materials dramatically reduce their strength and lifespan once they exceed a critical size. Compared to brittle materials such as glass and ceramics, viscoelastic hydrogels, as soft materials, exhibit a much greater length of flaw sensitivity length. Understanding the molecular mechanisms that govern flaw sensitivity in viscoelastic materials is crucial for optimizing their applications and designing tough materials. Herein, we use viscoelastic polyampholyte hydrogels as a model system to investigate how the hierarchical structure, including the transient network, primary network, and bicontinuous phase-separated structure, affects the flaw sensitivity length (*c*_c_). Our findings reveal that *c*_c_ is strongly influenced by the primary network but only weakly dependent on the transient network. Furthermore, we estimate the prefactor (1/*k*) in the relationship between *c*_c_ and the fractocohesive length (Γ/W*), given by *c*_c_ = (1/*k*)*Γ*/W*, which is derived from elastic fracture mechanics, for both viscoelastic and relatively elastic soft materials. Additionally, we identify a universal correlation between *c*_c_ and the crack blunting indicator—the ratio of fracture stress to Young’s modulus (*σ*_f_/*E*)—which is applicable to both tough elastic and viscoelastic materials. This study offers insights into the mechanisms governing flaw sensitivity in viscoelastic materials.

## Graphical abstract

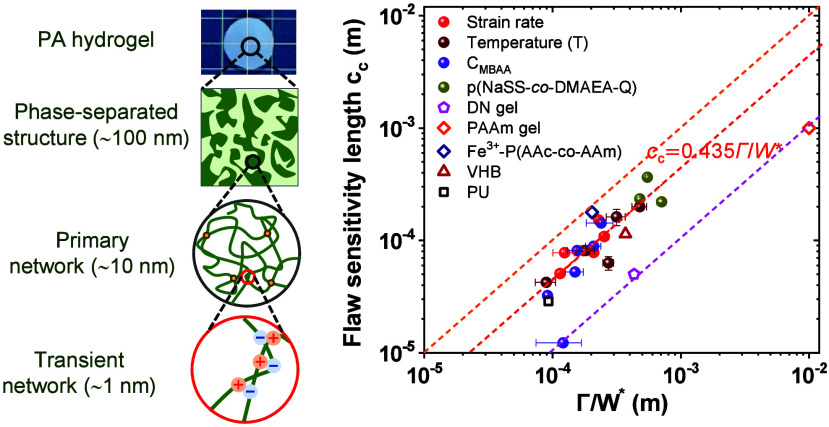

## Introduction

Hydrogels, composed of polymer networks swollen into water, have broad potential applications in tissue engineering,^1–3^ wound dressings,^4–6^ flexible electronics,^7^ soft actuators,^8–11^ etc. These applications usually require the hydrogels to exhibit combined exceptional mechanical properties, such as high strength, toughness, stretchability, self-healing, and fatigue resistance. Various strategies^12^ have been employed in the past two decades to create such tough hydrogels through various toughening mechanisms. For instance, based on the dynamic sacrificial bond mechanism, self-healing polyampholyte hydrogels,^13,14^ which exhibit a fracture toughness Γ (the energy required for crack growth per unit area under monotonic load) up to 4 kJ/m^2^ and a fatigue threshold (the critical energy required for crack extension along the crack plane under cyclic loads) up to 0.1 kJ/m^2^,^15,16^ were created; by incorporating dynamic sacrificial bond mechanisms into the double network principle,^17,18^ self-healing Ca-alginate/polyacrylamide hydrogel,^19^ which exhibits Γ up to 9 kJ/m^2^ and fatigue threshold around 0.05 kJ/m^2^,^20,21^ was created. The high toughness and fatigue resistance indicate that the hydrogels have strong crack resistance. However, the stretchability of these hydrogels was dramatically reduced by a pre-existent large flaw or crack, which should not be overlooked.

The reduction of stretchability by cracks or flaws is called flaw (crack) sensitivity,^22–24^ which causes the energy (*W*_b_) and stress (*σ*_f_) for rupturing a synthetic material much smaller than the theoretic values of that with non-defective structure by the relation *W*_b_ ∼ Γ/*c* (*c* > *c*_c_), where *c* is the crack or defect length and *c*_c_ is the critical length for materials becoming flaw sensitive.^25^ For instance, tough DN hydrogel without macroscale defect exhibits fracture stretch ratio *λ*_f_ = 9.59, *σ*_f_ = 1.04 MPa, and *W*_b_ = 5.9×10^6^ J/m^3^. In contrast, when a visible crack (0.4 mm) is introduced, the values decrease to *λ*_f_ = 3.38, *σ*_f_ = 0.61 MPa, and *W*_b_ = 9.2 × 10^5^ J/m^3^.^26^ *c*_c_ is an important material length that determines the fracture behavior. The phenomena of the scatter of rupture stress of uncut silica glass is enormous, while the scatter of rupture stress of uncut polymer hydrogels is small, and these are closely correlated to the length scale of *c*_c_. As the *c*_c_ value of silica glass is only on the order of 1 nm,^22^ any unintentional defects introduced during fabrication can cause stress concentration. In contrast, the *c*_c_ value of tough polymer hydrogels is typically larger than several hundreds of μm,^22,24^ and the unintentional defects are usually smaller than this length scale. Therefore, investigation of the physical mechanism related to the *c*_c_ of soft materials is extremely important for predicting the service life for practical applications and for guidance in creating new crack insensitive materials.

The *c*_c_ of several soft materials has been studied, such as elastic PAAm hydrogel,^27^ nonlinear elastic DN hydrogel,^26,28^ VHB and PU elastomers,^22^ bacterial cellulose hydrogels,^23^ and viscoelastic Fe^3+^ coordinated poly­(acrylic acid-*co*-acrylamide) (Fe^3+^-P­(AAc-*co*-AAm)) hydrogels.^29^ Almost all the studies have focused on whether the elastic fracture mechanics can predict *c*_c_ by the relation *c*_c_ ∼ Γ/W*, where W* is the energy per unit volume required to break the material without macroscopic cracks. The ratio Γ/W* is associated with crack-tip load-transfer length (or fractocohesive length), which represents the size of the region around the crack tip where the stress or strain concentration is eliminated.^22,25^ However, the molecular mechanism behind the *c*_c_ values of tough hydrogels, especially for viscoelastic hydrogels with a hierarchical structure, is not well understood.

In this study, we employ polyampholyte hydrogels (PA gels) as a model system to investigate the relationship between hierarchical structures and the flaw sensitivity length (*c*_c_). PA gels exhibit viscoelasticity due to ionic bonds and possess a hierarchical structure comprising a ∼1 nm scale transient network, a ∼10 nm scale primary network, and a ∼100 nm scale bicontinuous hard/soft phase-separated network.^30^ By systematically varying cross-linker content, monomer concentration, and chemical structure, we explore how these hierarchical structures influence *c*_c_. Furthermore, we tune the measurement strain rates and temperatures to explore the influence of the viscoelastic response on flaw sensitivity length *c*_c_. We estimate the prefactor (1/*k* ≈ 0.435) in the relationship for viscoelastic tough hydrogels,

1 $$c_{c}\approx \left(1/k\right)\Gamma /W^{*}$$

where this scaling originates from elastic fracture mechanics theory but is now adapted to viscoelastic systems. Furthermore, polarization testing reveals that the stress concentration zone expands when the flaw size exceeds *c*_0_ > *c*_c_, establishing a correlation between flaw sensitivity and crack blunting behavior. These findings provide fundamental insights into the fracture mechanics of viscoelastic tough hydrogels and offer guidance for optimizing their mechanical properties.

## Materials and Experiments

### Materials

Anionic monomer sodium *p*-styrenesulfonate (NaSS) [FUJIFILM Wako Pure Chemical Corporation], cationic monomer 3-(methacryloylamino)­propyltrimethylammonium chloride (MPTC) [Sigma-Aldrich Japan] and methyl chloride quarternized *N*,*N*-dimethylaminoethyl acrylate chloride (DMAEA-Q) [MT Aqua Polymer, Inc.], UV initiator α-ketoglutaric acid (α-keto) [Wako Pure Chemical Industries, Ltd.], and cross-linker *N*,*N*′-methylenebis­(acrylamide) (MBAA) [Wako Pure Chemical Industries, Ltd.] were used. The structures of the chemicals are shown in Figure S1. All chemicals were used as received, and Millipore deionized water was used for all of the experiments.

### Synthesis of PA Gel

The charge-balanced P­(NaSS-*co*-MPTC) hydrogels were synthesized using a one-step random copolymerization process, as detailed in our previous research.^13^ In short, the cationic monomer MPTC, anionic monomer NaSS, initiator α-keto, and cross-linker MBAA were prepared as a mixed aqueous solution, in which the total monomer concentration (*C*_m_ = [NaSS] + [MPTC]) was 2.1 M with [NaSS]/[MPTC] = 0.5205:0.4795, and the initiator was 0.25 mol % relative to the *C*_m_. The cross-linker MBAA content (*C*_MBAA_) was controlled from 0 to 2 mol % relative to the *C*_m_. The mixed aqueous solution was then injected into a sandwich-like sample cell with a spacer thickness of 1 mm and exposed to UV light (wavelength: 365 nm) for 11 h under an argon atmosphere. Following polymerization, the resulting gels (as-prepared PA gels) were immersed in a large volume of water (room temperature) for 2 weeks to dialyze the counterions and residual chemicals at room temperature. The dialysis water was exchanged daily. During the dialysis process, ionic bonds formed between randomly distributed oppositely charged ionic groups on polymer chains, leading to the collapse of the polyampholyte chains and the isotropic contraction of the PA gel. The dialysis processes also led to the formation of a phase separation structure arising from the interplay between the aggregation of polymer strands driven by ionic bonds and the cross-linker hindering such aggregation.^31^ The water equilibrium hydrogels are coded as P­(NaSS-*co*-MPTC)-*C*_m_-*C*_MBAA_. Water equilibrium gels were used in all the tests unless otherwise specified.

P­(NaSS-*co*-DMAEA-Q) hydrogels were prepared using a similar method. The total monomer concentrations were *C*_m_ = 1.7, 2.0, 2.5 M, and the molar ratio was NaSS: DMAEA-Q = 0.5125:0.4875. 0.1 mol % UV initiator and 0.1 mol % chemical cross-linker (relative to *C*_m_) were added to the monomer solutions to prepare hydrogels. After polymerization, the gels were dialyzed into water to remove the counterions and reach water equilibrium. The water equilibrium hydrogels are coded as P­(NaSS-*co*-DMAEA-Q)-*C*_m_-*C*_MBAA_. Water equilibrium gels were used in all of the tests unless otherwise specified.

### Uniaxial Tensile Test

Fracture tests were performed using a Shimadzu autograph (AG-X, Shimadzu) at a tensile speed of *ν* in a humidity chamber (>95%RH). Dumbbell-shaped samples with a thickness size of 0.8–1 mm (*t*), a height of 15 mm (*L*_0_) at the gauge of the sample, and a width of 6 mm (*w*) were used. As for flaw sensitivity tests, an initial deep notch (*c*_0_ = 0.01–1.5 mm) was made by a razor blade (combined with a micrometer screw) at the middle of the sample along the width direction (Figure 2a). Nominal stress σ was determined from the tensile force divided by the full cross-sectional area of the sample, and stretch ratio *λ* was determined from the ratio of the deformed sample length *L* to the initial length *L*_0_. The initial strain rate *ε̇* was determined by *ν*/*L*_0_. The critical stretch ratio for crack growth (λ_c_) is denoted as the stretch ratio at which the crack starts to extend. The work of extension for sample fracture (*W*_b_) is calculated from the stress–stretch curve of an unnotched sample when stretching to the λ_c_. The fracture stress σ_f_, λ_c_, and *W*_b_ were plotted with initial crack length *c*_0_. The extension work to fracture for unnotched samples is denoted as W*. Considering the gels may contain flaws, we took the initial crack length of unnotched sample from 0.5 μm (size of bicontinuous hard/soft phase separation) to 10 μm to represent uncertainty in the length of crack-like flaws.^29,32^ The critical crack length *c*_c_ for flaw sensitivity is determined from the intersections of extrapolation line of W* plateau and the log–log regression line of *W*_b_ versus *c*_0_ for the notched samples (Figure 2e).^25^ The fracture energy Γ of the samples with varying initial crack length *c*_0_ was calculated from the equation.^33^

2 $$\Gamma =\frac{6}{\sqrt{\lambda_{c}}}W_{b}c_{0}$$

To investigate the effect of the primary network on *c*_c_, tensile tests at a nominal strain rate (0.1 s^–1^) were performed on P­(NaSS-*co*-MPTC)-2.1-*C*_MBAA_ gels with *C*_MBAA_ varying from 0 to 2 mol % at 24 °C. To investigate the effect of the transient network, P­(NaSS-*co*-MPTC)-2.1-0.1 gels were tested under various conditions: strain rate 0.01–1 s^–1^ and temperature 8–72°C. P­(NaSS-*co*-DMAEA-Q)-*C*_m_-0.1 gels were used to investigate the effect of the chemical structure on *c*_c_. A nominal strain rate (0.1 s^–1^) and a constant temperature of 24 °C were used for stretching the P­(NaSS-*co*-DMAEA-Q)-*C*_m_-0.1 gels.

### Rheology Test

Rheology tests were performed by using an ARES rheometer (ARES-G2, TA Instruments). The rheometer was equipped with a force- and torque-balanced transducer, featuring a minimum oscillation torque of 0.05 μN·m and a torque resolution of 1 nN·m. A disk-shaped sample with 10 mm in diameter was glued between two parallel plates, with water filling the surrounding area during the tests. Frequency sweep measurements from 0.1 to 100 rad/s were performed at different temperatures ranging from 8 to 80 °C with a shear strain of 0.1%. The temperature was incrementally increased with an interval of 8 °C, and the temperature was equilibrated for 5 min before measurement. Master curves for storage modulus (*G*′), loss modulus (*G*″), and loss tangent tan δ over a wide frequency range were constructed at a reference temperature of 24 °C with the horizontal shift factor *a*_T_ and vertical shift factor *b*_T_ according to the time-temperature superposition (TTS) principle (Figures 1b,c).

### USAXS and SAXS Measurement

The Ultrasmall Angle X-ray Scattering (USAXS) measurements were performed at the BL19B2 beamline at SPring-8. The X-ray energy was 24 keV and the sample-to-detector distance was 42635 mm. The data acquisition time was 1 min per frame. SAXS measurement was performed at the BL19U2 beamline (Pilatus 2M detector), NCPSS, Shanghai, China. The energy of the X-ray was 12 keV, and the sample-to-detector distance was 5756 mm. The data acquisition time was 5 s per frame. All of the USAXS and SAXS data were analyzed by using Fit 2D software from the European Synchrotron Radiation Facility, and background scattering from the water/solution was subtracted from the data. The 2D USAXS and SAXS patterns were azimuthally integrated to yield 1D scattering profiles versus the scattering vector

3 q=4π⁡sin⁡θ/λ

where *q* is the module of the scattering vector, and λ and 2θ are X-ray wavelength and the scattering angle, respectively. The *d*-spacing of the phase-separated structure was estimated from the peak positions of 1D scattering profiles (*q*_peak_) by

4 $$d=2\pi /q_{\mathrm{peak}}$$

### Birefringence Observation

To observe the stress distribution during deformation and fracture, a homemade circularly polarized optical system was combined with a tensile machine (Figure S2). The sample was placed between two circularly polarized films (±45°) with a white LED light panel installed at the back side. A video camera was installed on the front side to record the changes in shape and birefringence color of the sample during tensile test. The optical retardation change of the sample during the tensile test was estimated by comparing the birefringence color to a Michel–Levy chart.^34^

## Results and Discussion

### Hierarchical Structure and Viscoelasticity of PA Gels

Ionic bonds between polyampholyte network strands form during the dialysis process to remove the counterions, which induces the formation of a bicontinuous phase-separated structure. Therefore, PA gels are composed of hierarchical structure, including a transient network (∼1 nm) due to reversible ionic bonds, a primary network (∼10 nm) due to chemical cross-linking and entanglements, and a bicontinuous phase-separated network (∼100 nm) due to hard and soft phase domains (Figure 1a). The bicontinuous phase-separated network structure is correlated to cross-linker content *C*_MBAA_, monomer concentration *C*_m_, chemical structure, and dialysis temperature to the water-equilibrium state.^16,30,31^ With increasing the *C*_MBAA_ from 0 to 0.5 mol %, appearance of the PA gels changed from cloudy to transparent with the *d*-spacing between the hard/soft domains decreasing from 690.1 to 52 nm for the P­(NaSS-*co*-MPTC)-2.1-*C*_MBAA_ gels. At *C*_MBAA_ > 0.5 mol %, no phase separation is observed because the cross-linker hinders aggregation of the polymer strands. The P­(NaSS-*co*-DMAEAQ)-*C*_m_-0.1 decreases from 154.7 to 82.4 nm with *C*_m_ increasing from 1.7 to 2.5 M, since both the chemical cross-linker and entanglement are against the polymer collapse caused by ionic bonds. The hierarchical structure results in a multiscale fracture process to dissipate a substantial amount of energy.^30^ At small to medium deformation, affine deformation occurs in the bicontinuous hard/soft phase network and ionic bonds break with polymer strands elongating. Further increasing the deformation, non-affine deformation occurs due to the damage in the hard phase network, but the soft phase network disperses the stress and prevents crack propagation. Finally, fractures in the soft phase network result in the catastrophic failure of the gel. The multiscale fracture processes not only contribute to high extensibility and toughness of the PA gels^30^ but also endow a delayed fatigue fracture.^15,16^

**Figure 1:**
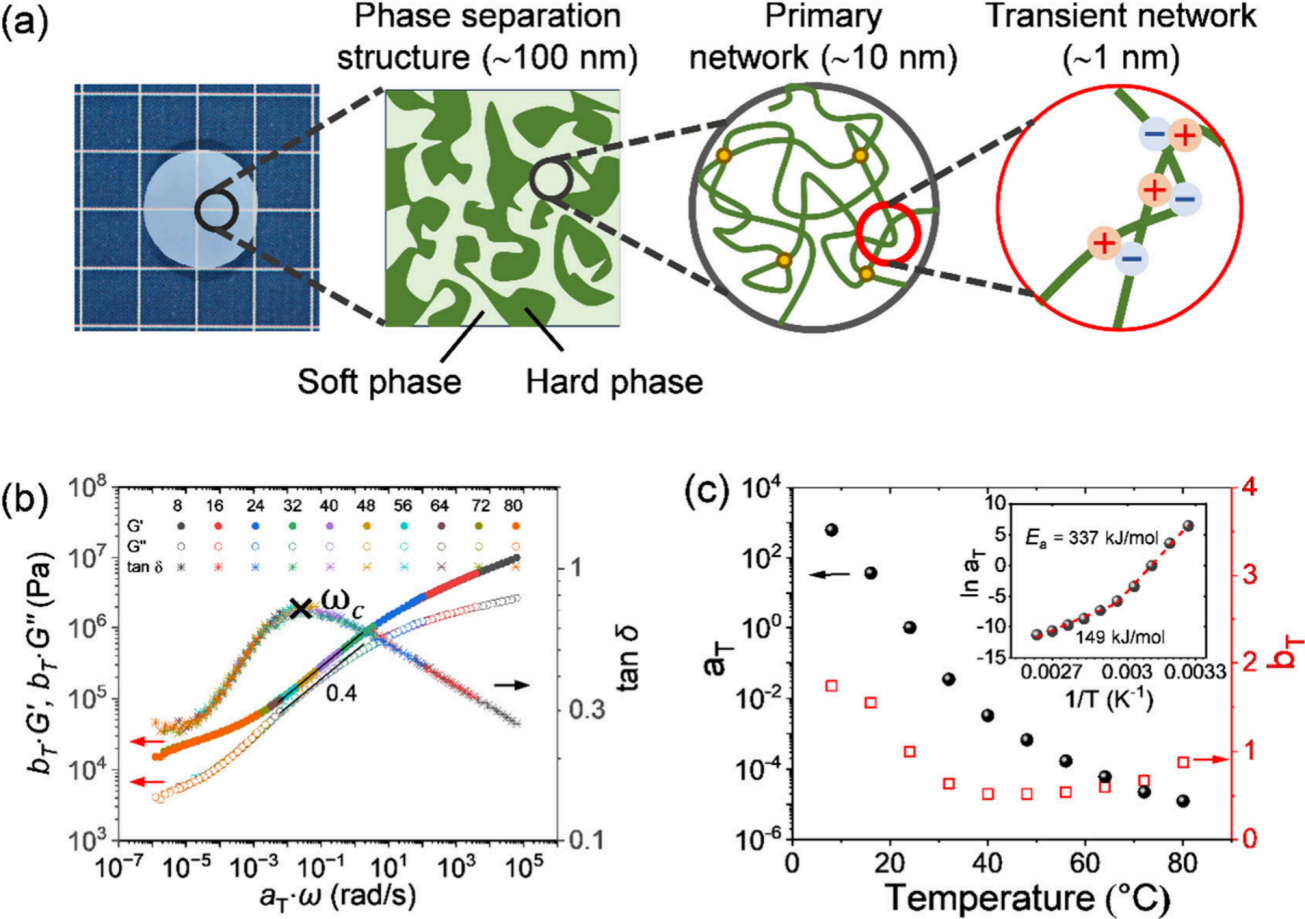
Hierarchical structure and rheology response of PA gels. P­(NaSS-*co*-MPTC)-2.1-0.1 is taken as an example. (a) Scheme of the hierarchical structure of PA gel, including ∼1 nm scale transient network, ∼10 nm scale primary network, and ∼100 nm scale phase-separated network. (b) Dynamic mechanical behavior. Constructed master curves for frequency dependence of storage modulus (*G*′), loss modulus (*G*″) and loss factor (tan δ) at a reference temperature of 24 °C by following the time–temperature superposition principle with horizontal shift factor *a*_T_ and vertical shift factor *b*_T_ (c). The inset in panel (c) shows that the *a*_T_ follows the Arrhenius equation *a*_T_ = *A* exp­(*E*_a_/*RT*), with the apparent activation energy *E*_a_ spanning a broad range from 149 to 337 kJ/mol. *ω*_c_ denotes the main viscoelastic relaxation frequency, from which we can obtain the main viscoelastic relaxation time τ for sticky Rouse behavior,^35^ by τ = 1/ω_c_.

PA gels also exhibit strong viscoelastic (Figure 1b,c) and rate-dependent tensile (Figure 4a) behaviors due to the existence of ionic bonds. Figure 1b shows that the frequency dependence of storage modulus (*G*′), loss modulus (*G*″) and loss factor (tan δ) obeys the time-temperature superposition (TTS) principle, with the corresponding horizontal (*a*_T_) and vertical (*b*_T_) shift factors presented in Figure 1c. *a*_T_ follows the Arrhenius equation

5 $$a_{T}=A\exp\left(E_{a}/RT\right)$$

where *A* is a constant, *E*_a_ is apparent activation energy, *R* is the ideal gas constant, and *T* is the absolute temperature. The temperature dependence of *a*_T_ shows that the activation energy of the PA gels spans a broad range from 149 to 337 kJ/mol (inset of Figure 1c), reflecting a wide distribution of ion association strengths. Notably, the upper limit of this activation energy approaches the dissociation energy of covalent C–C bonds (*E*_c‑c_ ≈ 350 kJ/mol), which may correspond to the ion associations stabilized by topological entanglement at temperatures below 40 °C. In contrast, at temperatures above 40 °C, the ion associations weaken with increasing temperature, resulting in a lower activation energy (*E*_a_ = 149 kJ/mol). This finding is consistent with the previous work.^13^ The vertical shift factor *b*_T_ was automatically calculated by rheometer software (e.g., TA Instruments TRIOS) based on experimental fitting results. This calculation typically incorporates: temperature dependence (*T*_ref_/*T*), density variations (*ρ*_ref_/*ρ*, the value keeps at *ρ*_ref_/*ρ* = 1) across temperatures, and a correction term *b*_*T*_^*δ*^ to account for deviations from theoretical predictions, i.e.,

6 $$b_{T}=b_{T}^{\delta }\frac{T_{ref}\rho_{ref}}{T\rho }$$

*b*_*T*_^*δ*^ is in the range 0.56–1.68. As a result, *b*_T_ varies from 0.52 to 1.74 across the temperature range 8–80 °C in the TTS analysis (Figure 1c). The slight fluctuations in the tan δ master curve observed between 32 and 48 °C likely originate from variations in the apparent activation energy *E*_a_. In the viscoelastic regime, both *G*′ and *G*″ exhibit a slope of ∼0.4, characteristic of sticky Rouse behavior. This value is slightly lower than the theoretical 0.5 slope predicted for homogeneous systems, arising from their phase-separated structure, as suggested by Cui and Gong.^36^

Under deformation, the viscoelastic hydrogels dissipate the stored strain energy over time.^37^ It is well-known that the viscoelastic dissipation results in remarkable enhancement of fracture energy Γ (the energy required per unit area of crack extension).^38,39^ As a result, Γ should couple physical processes at length and time scales. Among them, the crack tip load-transfer length ξ (ξ ∼ Γ/W*) is a crucial length scale for characterizing the soft and ductile materials, where W* is strain energy density for unnotch-sample failure. ξ represents the distance from the crack tip at which a characteristic load is transferred from the global mechanical fields to the failure processes, which is also related to the flaw-sensitivity by unspecified prefactors.^22,40^ At flaw size *c* ≪ ξ, the fracture behavior may be insensitive to *c* as the stress concentration is mitigated by dissipative processes occurring on the length scale ξ. In contrast, at flaw size *c* ≫ ξ, flaw-sensitive failure will occur. The scaling of ξ ∼ Γ/W* is derived from reversible elastic deformation materials. Recent studies^22,25,27^ on several soft materials reveal that its relationship with the flaw-sensitivity length *c*_c_ holds for a broad class of materials, as it does not specify whether the dissipation follows linear or nonlinear deformation behavior. The question is how is the flaw-sensitivity length *c*_c_ affected by the hierarchical structure and viscoelastic response in tough and self-healing hydrogels?

### Determining the Flaw Sensitivity Length *c*_c_

The flaw sensitivity length *c*_c_ was determined by uniaxial tensile strength for specimens with different initial crack lengths *c*_0_, as shown in Figure 2a,b. During the test, a video camera recorded the crack propagation behavior. The starting point of crack propagation was defined as the critical stretch ratio of crack propagation, *λ*_c_, and the area of the stress–stretch curve of the unnotched sample up to *λ*_c_ was defined as work to failure, *W*_b_ (Figure 2c). From uniaxial tensile test, we obtained the relationships of *λ*_c_ versus the initial crack length *c*_0_ (Figure 2d), *W*_b_ versus *c*_0_ (Figure 2e) and fracture stress σ_f_ versus *c*_0_ (Figure 2f). As an example, Figure 2 illustrates the estimation of *c*_c_ from the *λ*_c_, *W*_b_ and σ_f_ versus *c*_0_ plots for the P­(NaSS-*co*-MPTC)-2.1-0.1 gel. Considering potential flaws in the gels, we assumed an initial crack length ranging from 0.5 μm (corresponding to the size of bicontinuous hard/soft phase separation) to 10 μm for unnotched samples, representing the uncertainty in crack-like flaw dimensions. The critical crack length (*c*_c_) for flaw sensitivity was determined from the intersection points between the extrapolated line representing crack-like flaw uncertainty in unnotched samples and the log–log regression lines of λ_c_, *W*_b_, and *σ*_f_ versus *c*_0_ from notched samples.

**Figure 2:**
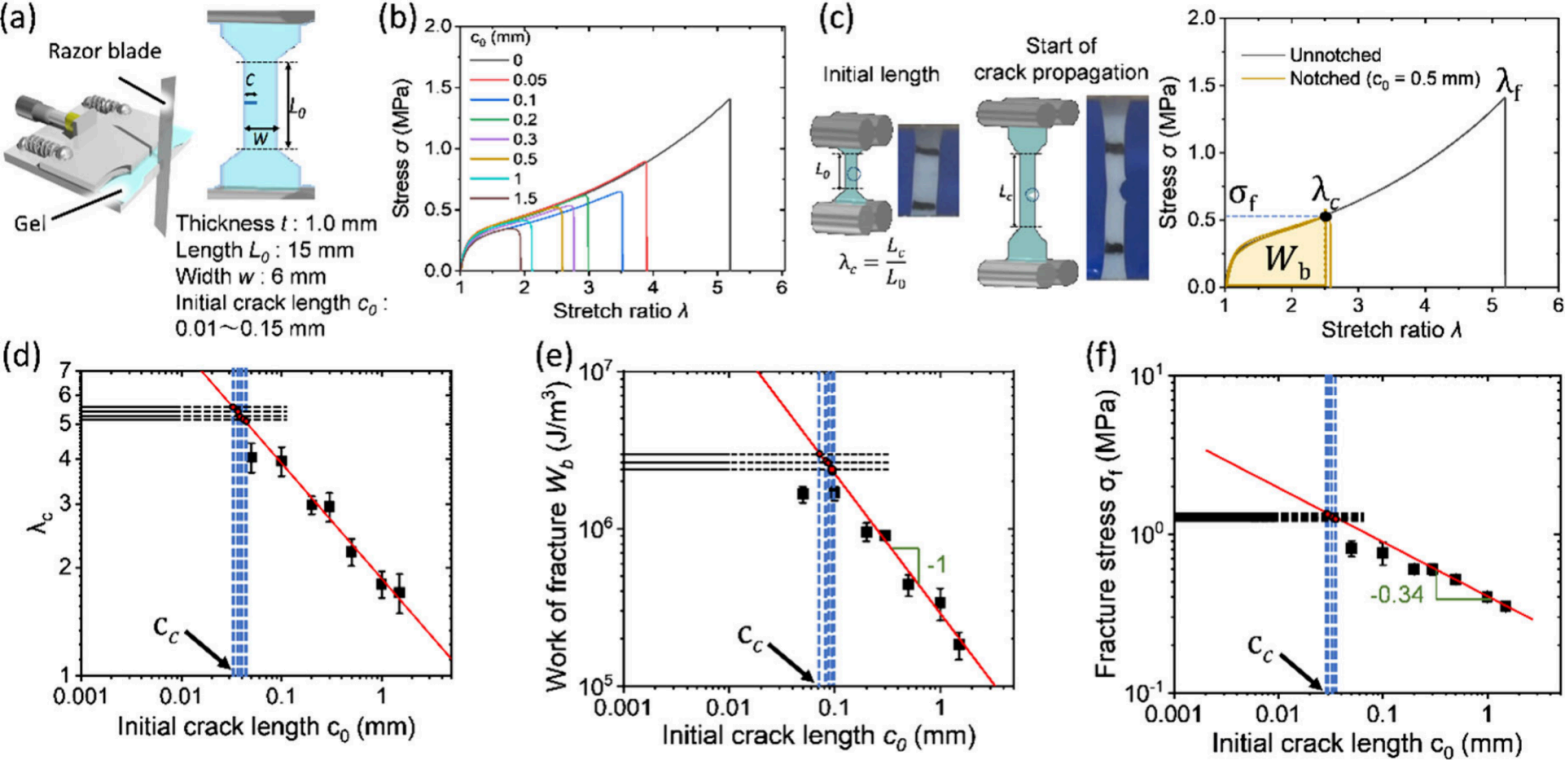
Determining the flaw sensitivity length *c*_c_. (a) Sample geometry and method to control the initial flaw (crack) length *c*_0_. (b) The stress–stretch curve of PA gel with varied initial crack length *c*_0_. P­(NaSS-*co*-MPTC)-2.1-0.1 is taken as an example. The experiments were conducted at a strain rate of 0.111 s^–1^ and a temperature of 24 °C. (c) Scheme for determining the onset of crack growth *λ*_c_, the work of fracture *W*_b_, and fracture stress *σ*_f_. (d) λ_c_ as a function of *c*_0_. (e) *W*_b_ as a function of *c*_0_. (f) Fracture stress, *σ*_f_ as a function of *c*_0_. In panels (d) to (f), the horizontal lines represent the values obtained from five measurements of unnotched samples, incorporating crack-like flaw uncertainties ≤10 μm, and dotted lines are the extrapolated line. The red line shows the log-log regression fit to the experimental data. *c*_c_ values were determined from the intersection of the horizontal dotted lines with the red fitting line. Error bars indicate the standard deviation based on five measurements.

The *c*_c_ estimated from *W*_b_ exhibits the largest value (0.081 mm), while *c*_c_ estimated from σ_f_ exhibits the smallest value (0.03 mm). *c*_c_ estimated from *λ*_c_ yields a value (*c*_c_ = 0.037 mm) that closely matches the one obtained from σ_f_. The reason for the discrepancies in *c*_c_ estimates from the three parameters remains unclear at present. However, all values fall within the same order of magnitude. According to the Griffith theory,^41^ for linear elastic materials, the σ_f_ has a relation with flaw size *c*_0_ by

7 $$\sigma_{f}∼{\left(\gamma /Ec_{0}\right)}^{1/2}$$

where γ is surface energy and *E* is Young’s modulus. In our work, we obtain σ_f_ ∼ *c*_0_^–0.34^, probably affected by the nonlinear viscoelasticity effect. From the *W*_*b*_ versus *c*_0_ plot, we obtain an approximate relationship *W*_*b*_ ∼ *c*_0_^–1^, which coincides with the nonlinear theory prediction.^25^

8 $$W_{b}≃\frac{\Gamma }{c_{0}}$$

In the following section, *c*_c_ obtained from *W*_b_ versus *c*_0_ plots will be used as a representative example to illustrate its correlation with the hierarchical structure. For reference, *λ*_c_ versus *c*_0_ plots and σ_f_ versus *c*_0_ plots for all the samples are shown in Figures S4, S6, and S9.

### Effect of Hierarchical Structure on *c*_c_

P­(NaSS-*co*-MPTC)-2.1-*C*_MBAA_ hydrogels were used for studying the effect of the primary network on *c*_c_. Figure 3a,b shows their appearance and tensile behaviors of unnotched gels. As cross-linker content *C*_MBAA_ increases, the gel changes from a non-transparent whitish appearance to transparent ones, which is associated with the phase-separated structure.^31^ As the cross-linker concentration increased from 0 to 2 mol %, the fracture stretch ratio *λ* of unnotched samples decreased significantly from 5.8 to 1.8, whereas the fracture stress *σ* showed only a slight change, decreasing from 1.5 to 1.3 MPa (Table 1). As for notched samples, they exhibit flaw sensitive behavior, as shown in Figure 2b and Figure S3.

**Table 1:** Phase-Separated Structure, Mechanical Properties, and Flaw-Sensitivity Length of PA Gels Fabricated Using Various Formulas

Sample name	Phase size (nm)	quasi-platform modulus (kPa)	Young’s modulus (kPa)	Unnotched sample fracture stress σ_f_ (MPa)	W* (MJ/m^3^)	Γ^a^ (kJ/m^2^)	Flaw sensitivity length *c*_c_ (mm)
P(NaSS-*co*-MPTC)-2.1-0	690.1	8.5	856.9	1.4	0.03	1.60	0.143
P(NaSS-*co*-MPTC)-2.1-0.1	247.8	18.2	1130.6	1.3	2.73	0.88	0.081
P(NaSS-*co*-MPTC)-2.1-0.3	88.1	28.9	1611.3	1.3	2.48	0.98	0.089
P(NaSS-*co*-MPTC)-2.1-0.5	51.9	42.7	1615.7	1.4	2.14	0.81	0.052
P(NaSS-*co*-MPTC)-2.1-1	38.9	75.7	2307.9	1.2	1.32	0.49	0.032
P(NaSS-*co*-MPTC)-2.1-2	40.4	97.1	4122.5	0.4	0.77	0.64	0.012
P(NaSS-*co*-DMAEA-Q)-1.75-0.1	154.7	7.9	62.8	0.5	1.12	0.78	0.221
P(NaSS-*co*-DMAEA-Q)-2.0-0.1	119.2	8.8	64.1	0.6	1.55	1.09	0.365
P(NaSS-*co*-DMAEA-Q)-2.5-0.1	82.4	13.9	52.8	0.4	1.13	0.68	0.234

a The fracture energy Γ is determined from the high plateau values observed in the Γ versus *c*_0_ curves when the initial crack length approaches *c*_0_ ≈ 1 mm (Figure S13). These measurements were conducted at a temperature of 24 °C and a strain rate of 0.111 s^–1^.

**Figure 3:**
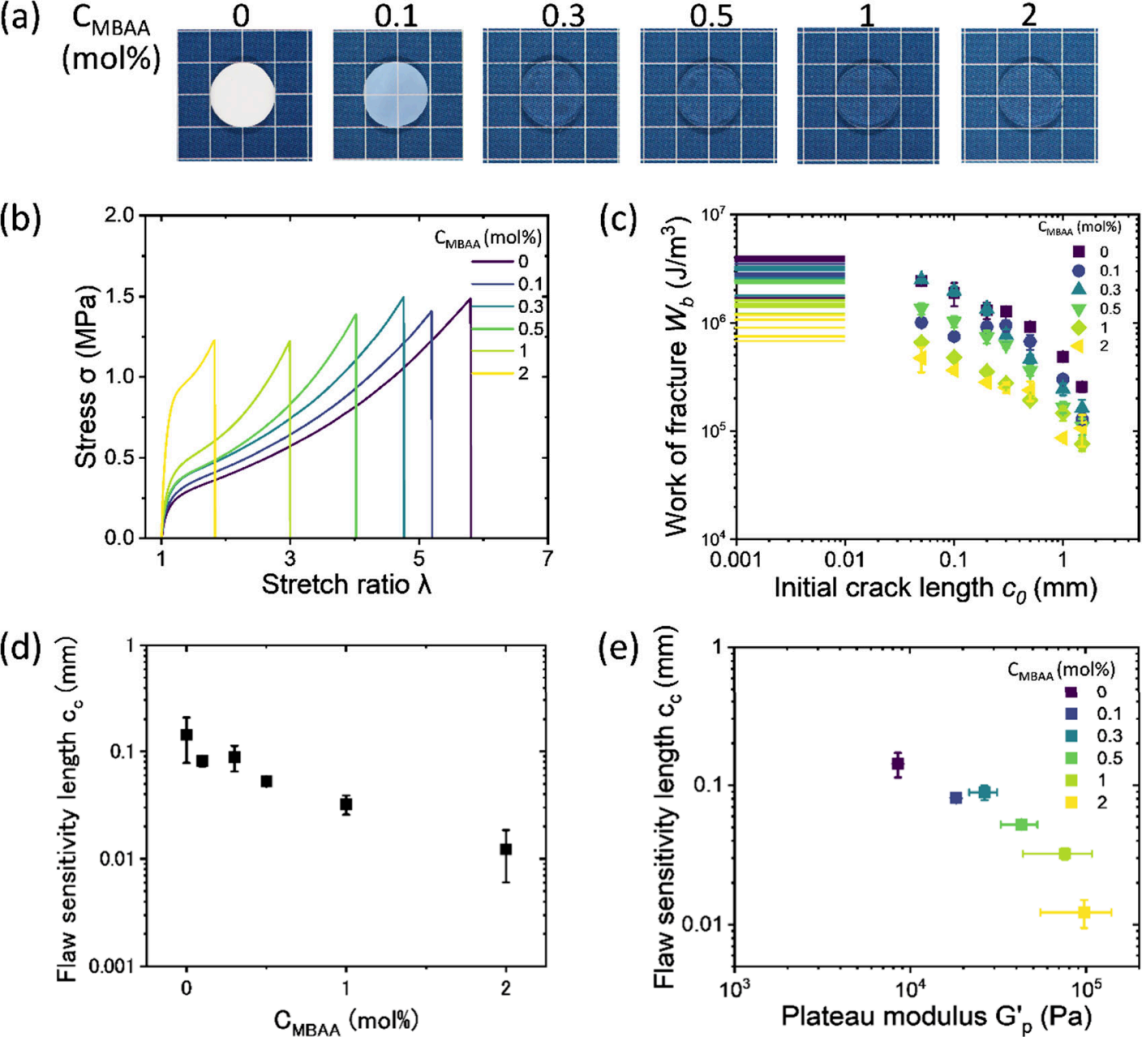
Effect of effective primary network on flaw sensitivity length *c*_c_. (a) Appearance of P­(NaSS-*co*-MPTC)-2.1-*C*_MBAA_ gels. (b) Tensile behavior of unnotched P­(NaSS-*co*-MPTC)-2.1-*C*_MBAA_ under a strain rate of 0.111 s^–1^ at 24 °C. (c) Relationship between *W*_b_ and *c*_0_ for P­(NaSS-*co*-MPTC)-2.1-*C*_MBAA_. Stress–stretch curves used to estimate *W*_b_ are shown in Figure S3. The horizontal lines indicate *W*_b_ values for samples without precuts (five measurements). (d) Flaw sensitivity length *c*_c_ as a function of *C*_MBAA_. (e) Correlation between *c*_c_ and the quasi-platform modulus (*G*′_p_) of P­(NaSS-*co*-MPTC)-2.1-*C*_MBAA_. Error bars represent the standard deviation from five measurements for *c*_c_ and at least two measurements for *G*′_p_.

From *W*_b_ versus *c*_0_ plots (Figure 3c), *c*_c_ was estimated. It should be noted that, for all sample composition, *W*_b_ has an approximate scaling relationship with *c*_0_, *W*_b_ ∼ *c*_0_^–1^, coinciding with the flaw-sensitive failure theory.^25^ *c*_c_ decreased by one-order-of-magnitude (100 μm to 10 μm) with increasing cross-linker concentration from 0 and 2 mol % (Figure 3d and Table 1). To verify the correlation between *c*_c_ and the mesh size of the polymer primary network, we plot *c*_c_ with the quasi-platform modulus *G*′_p_ from the constructed master curves for frequency dependence of storage modulus. *G*′_p_ is obtained from the storage modulus at an angular frequency near 10^–6^ rad/s as shown in Figure S3a. In this low angular frequency region, the contribution of ionic bonding to the shear modulus is expected to be almost negligible, so it is taken as the modulus derived from the effective primary network due to chemical cross-linking and permanent entanglement,^31^ by

9 $${G^{\prime }}_{p}=\nu_{e}kT$$

where *ν*_e_ is the volume density of effective primary network, having a correlation with mesh size *ξ*_0_ by

10 $$\nu_{e}={\xi_{0}}^{-3}$$

*k* is Boltzmann constant (J/K), and *T* is absolute temperature (K). *c*_c_ decreases with *G*′_p_ (Figure 3e) indicating that *c*_c_ decreases as the mesh size of the effective primary network *ξ*_0_ decreases.

To study how the transient network (formed by ionic bonds) and corresponding dynamic responses contribute to flaw insensitivity, we used P­(NaSS-*co*-MPTC)-2.1-0.1 hydrogel as an example, and conducted uniaxial tensile tests on both notched and unnotched samples at various strain rates from 10^–2^ to 1.1 s^–1^ (Figure 4a and Figures S5 and S6). From the *W*_b_ versus *c*_0_ plot (Figure 4b), we obtained *c*_c_. *c*_c_ is almost independent on the strain rates (Figure 4c), probably because the strain rate range used for the tests is too narrow and its effect on cross-linking points of transient network is weak.

**Figure 4:**
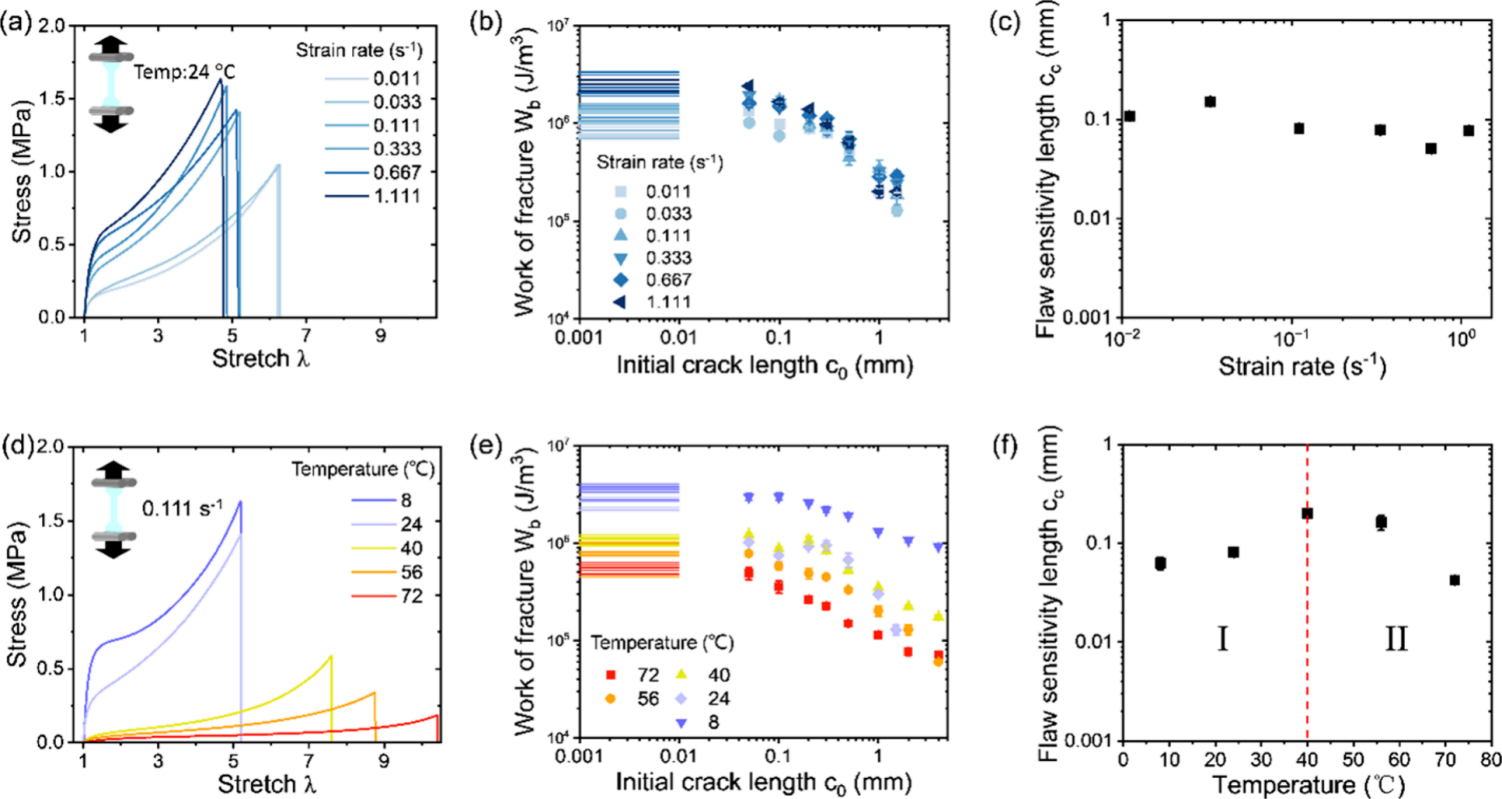
Effect of transient network on flaw sensitivity length *c*_c_. The P­(NaSS-*co*-MPTC)-2.1-0.1 hydrogel was taken as an example. (a) Tensile behavior of unnotched samples under different nominal strain rates. (b) Work of fracture *W*_b_ as a function of initial crack length *c*_0_ at various nominal strain rates. (c) Flaw sensitivity length *c*_c_ as a function of nominal strain rate. Measurements in (a)–(c) were conducted at a constant temperature of 24 °C. (d) Tensile behavior of unnotched samples under different temperatures. (e) Work of fracture *W*_b_ as a function of initial crack length *c*_0_ at various temperatures. (f) *c*_c_ as a function of tensile temperature. Measurements in panels d–f were conducted at a constant strain rate of 0.111 s^–1^. Error bars represent the standard deviation from five measurements. In (b) and (e), the horizontal lines indicate the *W*_b_ values of samples without precuts (five measurements).

To extend the observation time scale, we applied the time–temperature superposition principle. The temperature-controlled tests were conducted underwater. By matching the complex modulus G* versus angular frequency with Young’s modulus versus equivalent strain rates using shift factors (Figure S7), we showed that a wide range of equivalent strain rates (spinning eight orders of magnitude) could be achieved by varying the measurement temperatures from 8 to 72 °C. Although Figure S7 reflects the linear viscoelastic behavior, our previous work has shown that the mechanical performance under large deformation is closely correlated with this linear viscoelastic behavior. This is because the breakage of weak bonds is primarily governed by the thermal activation process, where the effect of forced debonding does not play a substantial role.^42,43^ Therefore, it is reasonable to assume that varying the tensile temperature adjusted the equivalent strain rates, spanning several orders of magnitude.

Figure 4d and Figure S8 show the stress–stretch curves of P­(NaSS-*co*-MPTC)-2.1-0.1 under tension at various temperatures, and the effect of tensile temperatures on *c*_c_ is shown in Figure 4e,f and Figure S9. As the temperature increases from 8 to 40 °C (regime I), *c*_c_ rises slightly from 0.063 to 0.2 mm, but then decreases to 0.042 mm as the temperature is further increased to 72 °C (regime II). In regime I, the weakening of the ionic bond (resulting in an increased transient network mesh size) leads to an increase in *c*_c_, though the detailed mechanism remains unclear. In regime II, however, the trend reverses. This may be because the contribution of ionic bonding becomes weaker, allowing the loosely cross-linked primary network to dominate the dynamic response. From the absence of a strict plateau at low frequencies (Figure S7), we can infer that slippage of poorly cross-linked chains occurs,^44^ particularly at 72 °C. This process may affect *c*_c_, leading to decrease in *c*_c_ with increasing temperature.

Phase separation is widely attributed to enhance the toughness of hydrogels due to the enhancement in crack resistance^12,45–48^ and load transfer length.^49,50^ We further investigated the effect of the phase-separated structure on *c*_c_. The phase structure was characterized using small angle X-ray scattering (SAXS) or ultrasmall angle X-ray scattering (USAXS). Figure 5 panels a and b show the 2D USAXS patterns and 1D scattering profiles of P­(NaSS-*co*-MPTC)-2.1-*C*_MBAA_ as an example. The scattering intensity decreases dramatically with increasing *C*_MBAA_. We estimated the *d*-spacing (*d*_0_) of hard/soft phase domains from the scattering peak position by eq 4 (Figure 5c), and it decreases dramatically from 690 to 52 nm with increasing *C*_MBAA_ from 0 to 0.5 mol %. This corresponds to a change in the appearance of the gel from cloudy to transparent (Figure 3a). Meanwhile, the scattering intensity *I*_peak_, which is correlated to the hard/soft phase contrast Δρ, shows the same decrease tendency with *C*_MBAA_ (Figure 5c) as that of *d*_0_. At *C*_MBAA_ ≥ 1.0 mol %, the scattering intensity decreases further without an observable scattering peak in the hydrogels. This behavior results from the high cross-linker concentration hindering aggregation of hydrophobic polymer backbones, thereby inhibiting the formation of phase-separated structures, consistent with our previous findings.^31^ For water equilibrium PA gels, *d*_0_ and Δρ should be intrinsically correlated (Δρ ∼ *d*_0_^2^) due to the competition between the driving force to promote phase separation (ionic and hydrophobic aggregation) and the resistance to prevent the phase separation (chemical cross-linking and permanent entanglement).^16,31^ To explore the universality of the effect of structure on *c*_c_, PA gels with different chemical structure, P­(NaSS-*co*-DMAEA-Q)-*C*_m_-0.1, were also fabricated, and their SAXS results, stress–stretch curves, and *c*_c_ results are shown in Figure S10. It shows that *c*_c_ depends little on *C*_m_, even though *I*_peak_ varies. This may be attributed to the fact that, compared with changing *C*_MBAA_, altering *C*_m_ from 1.75 to 2.5 M does not dramatically affect the effective cross-linking, because it can slightly increase entanglement but not change chemical cross-linking.^16,31^

**Figure 5:**
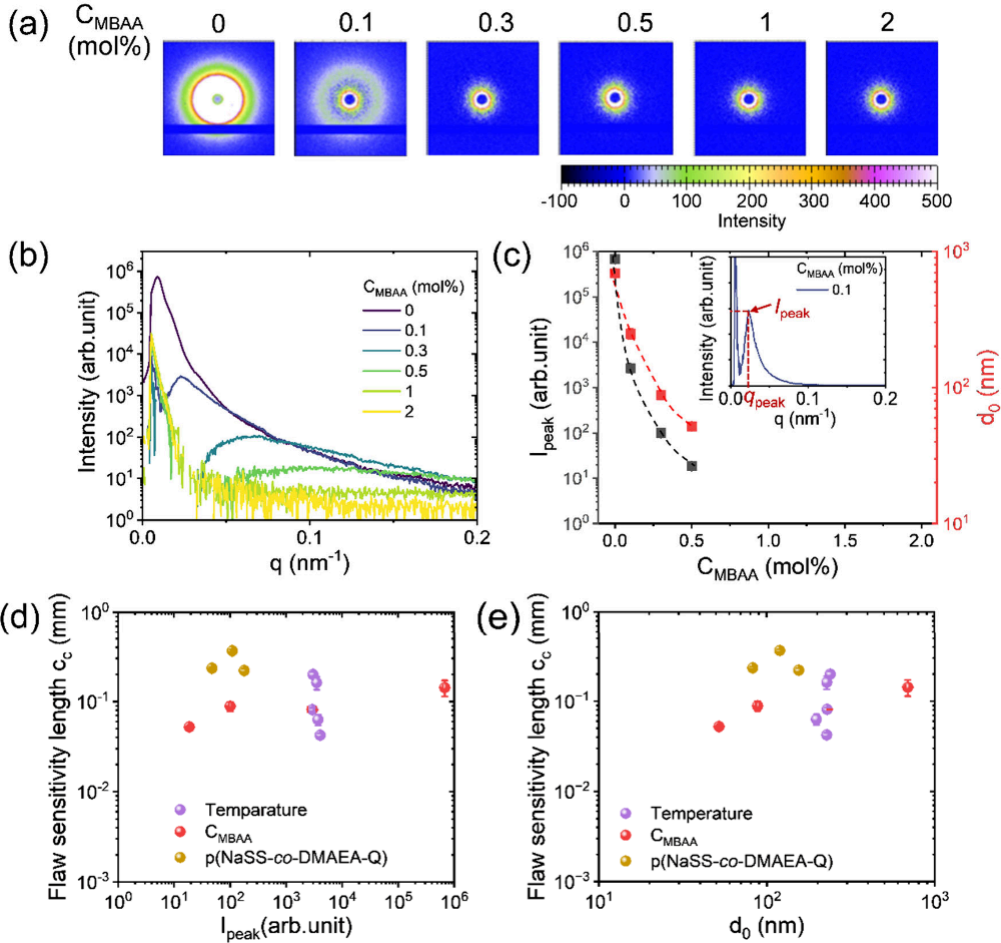
Effect of phase-separated structure on flaw sensitivity length *c*_c_. (a) Two-dimensional USAXS patterns of P­(NaSS-*co*-MPTC)-2.1-*C*_MBAA_ gels. (b) 1D scattering profiles of P­(NaSS-*co*-MPTC)-2.1-*C*_MBAA_. (c) Peak scattering intensity *I*_peak_ and *d*-spacing between hard or soft domains *d*_0_ as a function of *C*_MBAA_ for P­(NaSS-*co*-MPTC)-2.1-*C*_MBAA_. The inset illustrates the method for determining *I*_peak_ and peak position *q*_peak_. *d*_0_ is calculated by eq 4. (d)–(e) *c*_c_ as a function of *I*_peak_ (d) and *d*_0_ (e) for P­(NaSS-*co*-MPTC)-2.1-*C*_MBAA_, P­(NaSS-*co*-DMAEA-Q)-*C*_m_-0.1, and P­(NaSS-*co*-MPTC)-2.1-0.1 measured at temperatures ranging from 8 to 72 °C.

The phase separation structures of P­(NaSS-*co*-MPTC)-2.1-0.1 at varied temperatures were characterized by SAXS, and the results are shown in Figure S11. It is important to note that comparing the scattering intensity across different sets of data (USAXS and SAXS) is not meaningful. This is because the scattering intensity is influenced by numerous factors, including incident beam intensity, exposure time, etc. But the *I*_peak_ is an important parameter correlated to the phase contrast in the phase-separated PA system,^31^ by

11 $$I_{\mathrm{peak}}∼v_{s}v_{h}{\left(\Delta \rho \right)}^{2}$$

where *v*_h_ and *v*_s_ are the volume fraction of hard phase and soft phase, respectively, and Δρ is the density difference between them. Fortunately, the phase size *d*_0_ should not change using varied measurement setups, and the PA gels follow the intrinsic correlation *I*_peak_ ∼ *d*_0_^4^ irrelevant to the chemical structures.^31^ Therefore, we shifted the *I*_peak_ obtained from USAXS and SAXS results based on *I*_peak_ ∼ *d*_0_^4^ to normalize the *I*_peak_ to the same measurement condition (Figure S12).

Based on fracture mechanics theory,^22,25^ *c*_c_ shares the same expression formula as the load transfer length ξ (or *c*_c_) ∼ *Γ*/W*, with a prefactor that will be introduced later. A larger phase domain size and stronger phase contrast are expected to result in a larger load transfer length and *c*_c_. Figure 5 panels d and e show that for the P­(NaSS-*co*-MPTC)-2.1-*C*_MBAA_ series, where *I*_peak_ ranges from 10^2^ to 10^6^ arb unit and the phase-separated domain size *d*_0_ varies from 88 to 690 nm, *c*_c_ only increases slightly from 0.09 to 0.14 mm. However, P­(NaSS-*co*-MPTC)-2.1-0.1 across varying temperatures shows that, although *d*_0_ (≈230 nm) remains nearly unchanged with temperature, the variation in *c*_c_ (from 0.04 to 0.2 mm) is more pronounced than in the P­(NaSS-*co*-MPTC)-2.1-*C*_MBAA_ series. Additionally, P­(NaSS-*co*-DMAEA-Q)-*C*_m_-0.1, with *d*_0_ ranging from 82 to 155 nm, exhibits a similar *c*_c_ value (∼0.2 mm) to that of P­(NaSS-*co*-MPTC)-2.1-0, which has a much larger *d*_0_ of 690 nm (Figure 5d,e). These findings contradict the initial expectation, suggesting that *c*_c_ is not primarily governed by the phase-separated structure. It should be noted that the phase-separated structure itself is closely correlated with the polymer network’s mesh size, chemical structure, and thermal motion of the polymer network in the PA system. This combined effect may complicate the influence of the phase-separated structure on *c*_c_.

### *c*_c_ versus Γ/W* and σ_f_/*E*

The *c*_c_ of several materials has been well described based on elastic fracture mechanics theory, *c*_c_ ∼ Γ/W* with a prefactor 1/*k* (see eq 1).^24,26,29,51^ Here, *k* is a dimensionless number calculated by solving the boundary value problem of the elasticity. For a material with linear elasticity, *k* is 2π for a center-cracked sample and 2π(1.122)^2^ for an edge-cracked sample.^26^ For nonlinear materials, the specific form of *k* is more complex. We attempt to apply this mechanics theory to explain the *c*_c_ values of viscoelastic tough PA gels with hierarchical structures. To the best of our knowledge, current interpretations of *c*_c_ for tough hydrogels are primarily derived from the elastic fracture mechanics theory. However, there is a lack of systematic studies to examine whether these interpretations hold true under varying measurement conditions and material formulations, thereby verifying the scaling of *c*_c_ ∼ Γ/W*. For comparison, we include results of other soft materials, such as double network hydrogel (DN gel),^26^ polyacrylamide hydrogels (PAAm gel),^27^ and Fe^3+^ coordinated poly­(acrylic acid-*co*-acrylamide) hydrogel,^29^ as well as VHB and polyurethane (PU)^22^ on the same *c*_c_ versus Γ/W* plot.

We found that the fracture energy Γ of PA gels, calculated using eq 2,^33^ for single-edge crack test, depends on the crack length *c*_0_ (Figure S13). For samples measured at different temperatures, Γ consistently increases with *c*_0_. For sample series with varying strain rates and chemical cross-linker content (*C*_MBAA_), Γ increases with *c*_0_ when *c*_0_ < 1 mm, then reaches a high plateau as *c*_0_ increases. Note that, under the measurement conditions used here, all PA hydrogels exhibit *c*_c_ < 1 mm. These results suggest that this expression used for calculating the fracture energy may not work well for large-deformation hydrogels, as reported by Long and Hui.^33^ Here, we use fracture energy Γ estimated at *c*_0_ ≈ *c*_c_ and at *c*_0_ = 1 mm to calculate the Γ/W* and evaluate the validity of the scaling *c*_c_ ∼ Γ/W* for viscoelastic tough hydrogels. As shown in Figure 6a, when Γ is estimated at *c*_0_ ≈ *c*_c_, *c*_c_ follows *c*_c_ ∼ Γ/W* with a prefactor 1/*k* = 0.435. Intriguingly, almost all viscoelastic tough materials exhibit a similar 1/*k* value of 0.435, although the physics meaning of 1/*k* = 0.435 remains unclear at present. There is one exception: the highly cross-linked PA gel with *C*_MBAA_ = 2 mol %. Its *c*_c_ follows the same relationship as the double-network (DN) gel and PAAm gel, *c*_c_ ∼ Γ/W* with a prefactor 1/*k* = 0.104, which is close to 2π(1.122)^2^. This may be because these gels are relatively elastic, with the contribution of chemical cross-linking being greater than that of physical bonds.

**Figure 6:**
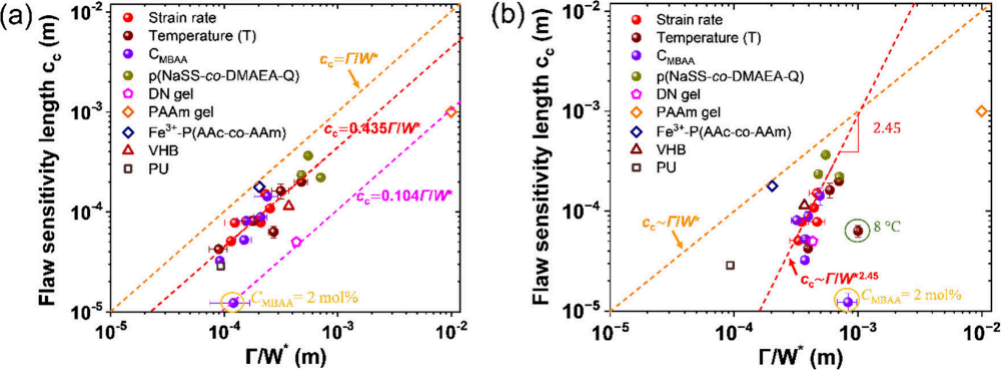
Relationship between *c*_c_ and Γ**/** W* for various soft materials. The strain rate, measurement temperature, *C*_MBAA_, and P­(NaSS-*co*-DMAEA-Q) data are from this study. (a) Γ/W* values obtained using Γ at *c*_0_ ≈ *c*_c_. (b) Γ/W* values obtained using Γ at *c*_0_ = 1 mm. Error bars represent the standard deviation from five measurements. The green-circled point represents P­(NaSS-*co*-MPTC)-2.1-0.1 measured at 8 °C, while the orange-circled point represents P­(NaSS-*co*-MPTC)-2.1-2.0. Data for DN gel,^26^ PAAm gel,^27^ and Fe^3+^-P­(AAc-*co*-AAm)^29^ were extracted from literature. *c*_c_ values of VHB and PU were estimated from ref 22.

Typically, the fracture energy of a material is considered an intrinsic property and should not depend on the crack length. Here, we also use Γ estimated at *c*_0_ = 1 mm, which reaches a *c*_0_-independent plateau under most measurement conditions. Figure 6b shows that the scaling of *c*_c_ versus Γ/W* for PA gels is approximately

12 $$c_{c}∼{\left(\Gamma /W^{*}\right)}^{2.45}$$

rather than *c*_c_ ∼ Γ/W*, except for two cases: the gel with a high cross-linker content *C*_MBAA_ = 2 mol %, and the gel with *C*_MBAA_ = 0.1 mol % gel measured at a low temperature of 8 °C. If the Γ value estimated at *c*_0_ = 1 mm is reliable (the total sample width is 6 mm), these results here suggest that nonlinear elastic fracture mechanics theory may not well explain the fracture behavior of viscoelastic soft materials with hierarchical structures.

Since crack growth is closely correlated to stress concentration, we used a homemade circular polarizing optical system^34^ to investigate the birefringence around the crack tip. The butterfly-shaped birefringence patterns confirm the existence of a yielding region with viscoelastic dissipation (Figure 7a,b). No butterfly-shaped birefringence pattern is observed in samples without an initial crack. Additionally, when the initial crack length *c*_0_ < *c*_c_, there is little change in birefringence color around the crack tip compared to the bulk material, indicating almost no stress concentration in this case. As the initial crack length *c*_0_ increases to *c*_0_ > *c*_c_, an obvious stress concentration is observed. These phenomena hold true for both lightly and densely cross-linked gels (Figure 7a,b). This suggests a correlation between the flaw sensitivity length and stress concentration. The results are consistent with the stress distribution observed below and above the flaw sensitivity lengths of bacterial cellulose hydrogel.^23^

**Figure 7:**
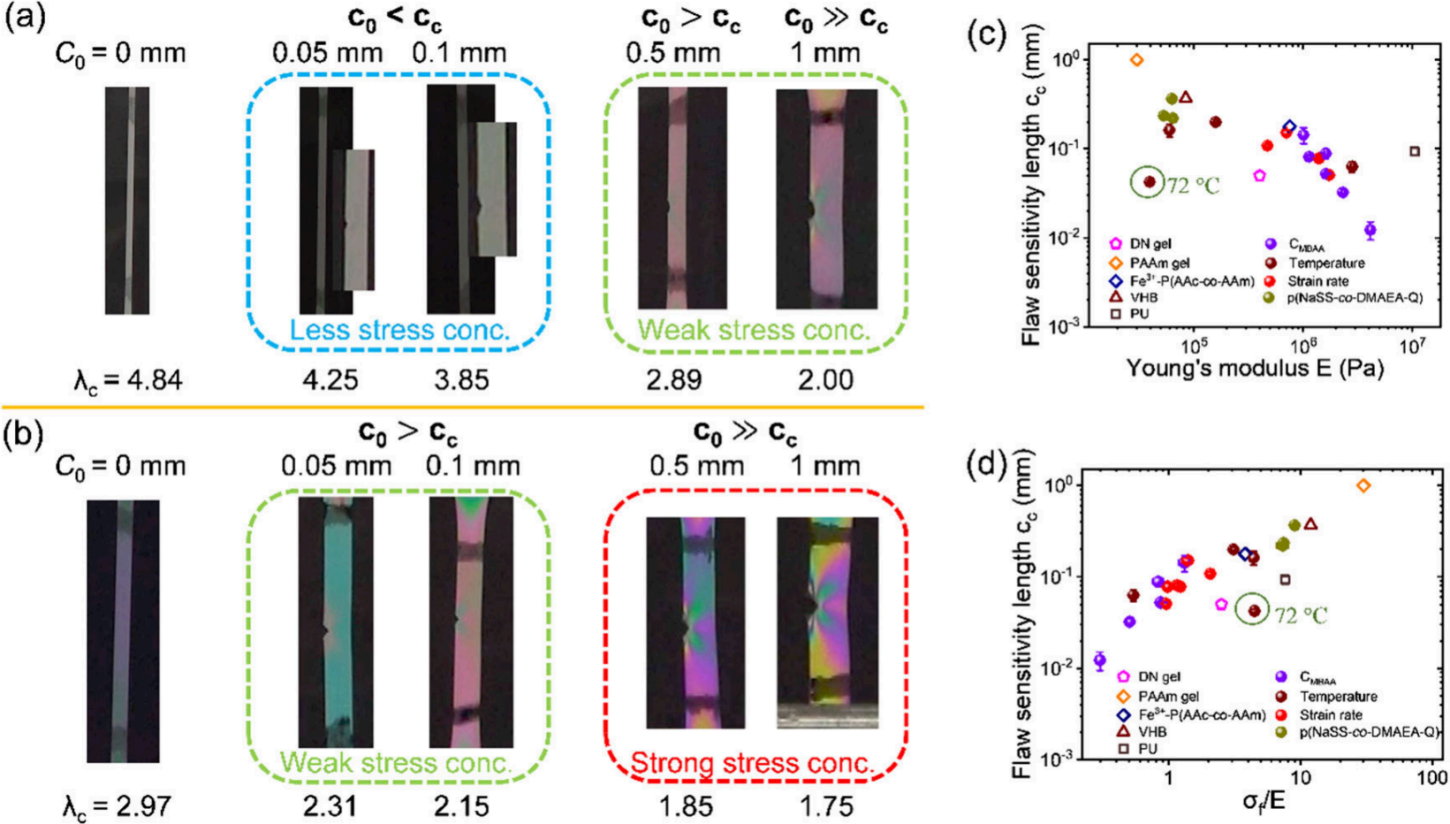
Relationship between *c*_c_ and crack blunting. (a,b) Birefringence images of P­(NaSS-*co*-MPTC)-2.1-0.1 (a) and P­(NaSS-*co*-MPTC)-2.1-1.0 (b) captured at λ_c_ for different initial crack lengths: *c*_0_ = 0*, c*_0_ < *c*_c_ and *c*_0_ > *c*_c_. (c) *c*_c_ as a function of Young’s modulus *E*. (d) *c*_c_ as a function of the ratio of fracture stress σ_f_ to Young’s modulus *E*. The green-circled point represents P­(NaSS-*co*-MPTC)-2.1-0.1 measured at 72 °C.

Figure 7 panels a and b also show that the lightly cross-linked P­(NaSS-*co*-MPTC)-2.1-0.1 exhibits a much blunter crack tip compared to the densely cross-linked P­(NaSS-*co*-MPTC)-2.1-1.0 at λ_c_. The P­(NaSS-*co*-MPTC)-2.1-1.0 has a higher modulus than P­(NaSS-*co*-MPTC)-2.1-0.1. To determine whether crack blunting and flaw sensitivity length *c*_c_ are primarily influenced by modulus, we plotted *c*_c_ as a function of Young’s modulus *E* (Figure 7c). *c*_c_ values of PA gels, which vary in cross-linker content *C*_MBAA_, monomer concentration *C*_m_, chemical structure, and measurement temperature, show a notable decreasing trend with increasing Young’s modulus (except at high measurement temperatures of 72 °C, which may be caused by chain slippage at high temperatures). However, when data from other tough soft materials, such as DN gel,^26^ PAAm gel,^27^ Fe^3+^-P­(AAc-*co*-AAm),^29^ and VHB and PU,^22^ are included in the graph, the trend becomes slightly disrupted.

In 2002, Hui et al.^52^ reported that crack blunting occurs when the strength of a soft material exceeds its elastic modulus, as demonstrated through large-deformation finite-element model simulations. This finding was further supported by their experiments on polyvinylbutyral (PVB) elastomers and later by experiments on PA gels conducted by Gong’s group.^34^ To explore the correlation between flaw sensitivity and crack blunting, we plotted *c*_c_ as a function of the ratio of fracture stress to Young’s modulus *σ*_f_/*E*. An increase in *c*_c_ is observed with an increase in *σ*_f_/*E*, and this correlation holds true for nearly all amorphous tough hydrogels studied for *c*_c_ to date (Figure 7d). This suggests that flaw sensitivity may be predicted by *σ*_f_/*E* and could potentially be a universal relationship across various tough soft materials regardless of their chemical structures or measurement conditions.

We also observed a strong correlation between *c*_c_ and fracture stress ratio (λ_f_) of PA gels without an initial crack (Figure S14)­

13 $$c_{c}∼{\lambda_{f}}^{1.56}$$

which may help explain the molecular mechanism underlying *c*_c_. Assuming that λ_f_ represents the maximum stretch ratio, it should correlate with the ratio of the contour length of one polymer strand *L*_c_ to the end-to-end distance of the undeformed strand *R*_0_, λ_f_ = *L*_c_/*R*_0_. For polyampholyte hydrogels, the network strands collapse in globule conformation due to aggregation induced by ionic bonds.^14,53^ Consequently, we have

14 $$R_{0}=b{N_{\mathrm{eff}}}^{1/3}$$

where *b* is monomer length and *N*_eff_ is the monomer number on one effective network strand.^16,53^ The contour length of one polymer strand is given by *L*_c_= *bN*_eff_. Therefore, in PA hydrogels, the maximum stretch ratio approaches the scaling

15 $$\lambda_{f}∼{N_{\mathrm{eff}}}^{2/3}$$

By combining the eq 13 and eq 15, we derive an approximate scaling *c*_c_ ∼ *N*_eff_. It should be noted that the *N*_eff_ represents the number of monomers on one effective network strand, accounting for both chemical cross-linking and effective strong ionic cross-linking. This finding coincides with above results, which indicates that *c*_c_ is correlated with the chemical cross-linker content and the equivalent strain rate (higher equivalent strain rate results in more effective strong ionic cross-linking).

## Conclusion

Using polyampholyte hydrogels as a model system, we investigated the effects of hierarchical structures—including the primary network, transient network, and bicontinuous hard/soft phase-separated network, on the flaw sensitivity length *c*_c_. By systematically varying chemical cross-linker content, monomer concentration, chemical structure, strain rates, and measurement temperatures, we found that *c*_c_ is strongly influenced by the primary network but only weakly dependent on the transient network within our observation time scale. Both the primary network and effective strong ionic bonding reduce *c*_c_ by decreasing the monomer number per effective strand. However, a clear correlation between *c*_c_ and the mesoscale bicontinuous hard-soft phase-separated network has not yet been established, likely due to its coupling with both the primary and transient networks. Further studies are needed to decouple these hierarchical structures, potentially using methods such as osmotic pressure control^54^ to tune the phase-separated structure.

Additionally, the load-transfer length or fractocohesive length, scaled as Γ/W* from elastic fracture mechanics theory, is applicable to explain the *c*_c_ values of viscoelastic PA hydrogels when the fracture energy Γ is estimated at *c*_0_ *= c*_*c*_ in single-edge crack test. We established that the prefactor 1/*k* = 0.435 in eq 1 exhibits universality, being material-independent for tough hydrogels and consistently higher than values for elastic materials. However, when Γ is estimated in a *c*_0_-independent regime, scaling *c*_c_ ∼ Γ/W* no longer holds. Instead, a new relationship (eq 12) emerges. Furthermore, we reveal that the flaw sensitivity length *c*_c_ correlates with the ratio of fracture stress to Young’s modulus, *σ*_f_/*E*, a parameter characterizing crack blunting behavior.^52^ This correlation appears to be universal for both elastic and viscoelastic tough hydrogels. The molecular mechanisms underlying flaw sensitivity and the novel physical correlations revealed in this study provide new insights into the flaw sensitivity in viscoelastic materials.

## Supplementary Material



## Notes

Chemical structures, diagram of birefringence observation, storage modulus, stress–stretch ratio curves, crack sensitivity, tensile behavior, matching the Young’s modulus from tensile tests with the linear rheology master curve, appearance and SAXS measurement, effect of temperature on phase-separated structure, correlation between SAXS/USAXS peak intensity and *d*-spacing, Γ as a function of initial crack length, and *c*_c_ as a function of fracture stretch (PDF)
