# Supplementary material for: Flaw Sensitivity of Tough and Self-Healing Hydrogels with Hierarchical Structure
Source: Polym Sci Technol. 2025 Apr 23;1(5):476–87. doi: 10.1021/polymscitech.5c00018 (PMC13052672; doi:10.1021/polymscitech.5c00018)
Supplement: Supplementary file 1 [file ps5c00018_si_001.pdf]

Supporting Information for

Flaw Sensitivity of Tough and Self-Healing Hydrogels with Hierarchical Structure

Reina Watanabe[1], Haruna Tsuchibora[1], Ryuji Kiyama[2,3], Kunpeng Cui[4,5], Xueyu Li[2,6]**

[1] Laboratory of Soft & Wet Matter, Division of Soft Matter, Graduate School of Life Science, Hokkaido University, Sapporo 060-0810, Japan

[2] Laboratory of Soft & Wet Matter, Faculty of Advanced Life Science, Hokkaido University, Sapporo 001-0021, Japan

[3] Laboratoire de Sciences et Ingénierie de la Matière Molle, CNRS, ESPCI Paris, PSL Research University, 10 rue Vauquelin, 75005 Paris, France

[4] Department of Polymer Science and Engineering, University of Science and Technology of China, Hefei 230026, China

[5] Institute for Chemical Reaction Design and Discovery (WPI-ICReDD), Hokkaido University, Sapporo 001-0021, Japan

[6] Hefei National Research Center for Physical Sciences at the Microscale, University of Science and Technology of China, Hefei 230026, China

*Email: kpcui@ustc.edu.cn; lixueyu@ustc.edu.cn

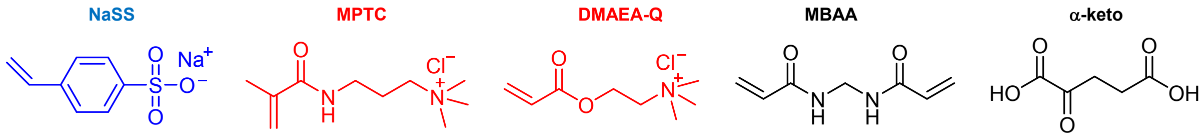

Figure S1. Structure of the chemicals used for synthesizing polyampholyte (PA) hydrogels.

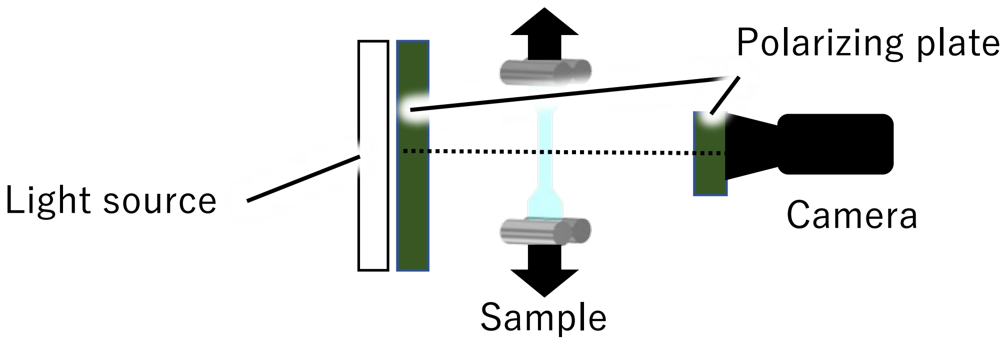

Figure S2. Schematic diagram of birefringence observation in tensile and fracture tests.

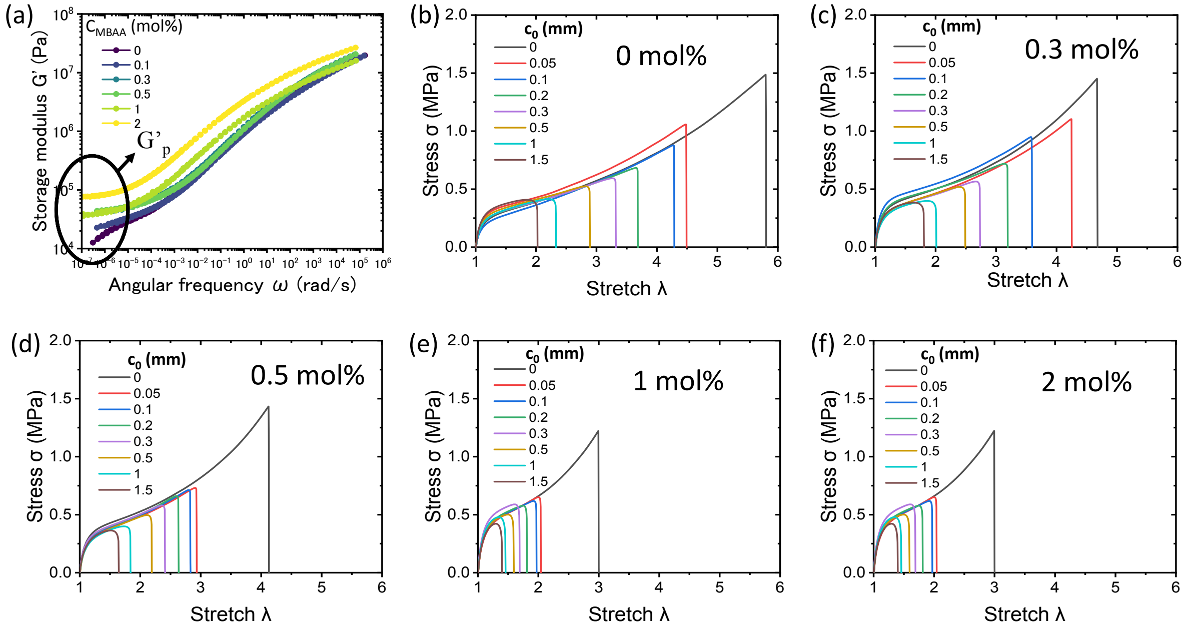

Figure S3. Mechanical performance of P(NaSS-*co*-MPTC)-2.1-C_{MBAA}. (a) Storage modulus *G*' as a function of angular frequency (ω) for P(NaSS-*co*-MPTC)-2.1-C_{MBAA} (master curve at a reference temperature of 24 ℃ by following the time-temperature superposition principle). (b-f) Nominal stress–stretch ratio curves of hydrogels with different initial crack lengths c_0 for P(NaSS-*co*-MPTC)-2.1-0 (b), P(NaSS-*co*-MPTC)-2.1-0.3 (c), P(NaSS-*co*-MPTC)-2.1-0.5 (d), P(NaSS-*co*-MPTC)-2.1-1 (e), and P(NaSS-*co*-MPTC)-2.1-2 (f). For (b) to (f), a strain rate of 0.111 s^{-1} was applied, and the measurement temperature was 24 °C.

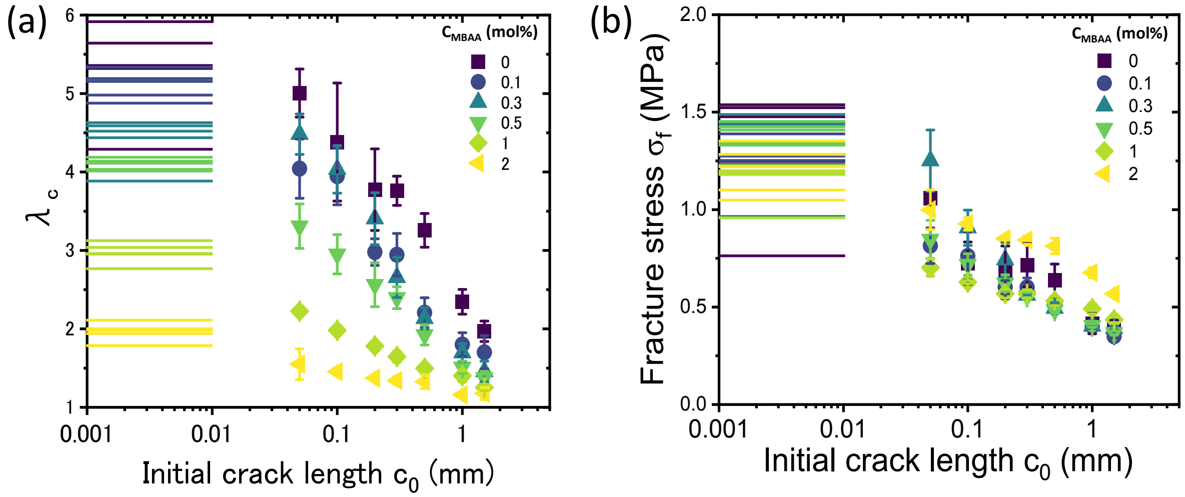

Figure S4. Crack sensitivity of P(NaSS-*co*-MPTC)-2.1-C_{MBAA} estimated by the critical stretch ratio for crack propagation (λ_c) and fracture stress (σ_f). (a) λ_c as a function of initial crack length c_0. (b) σ_f as a function of c_0. Horizontal lines represent values for samples without precuts (five measurements). A strain rate of 0.111 s^{-1} was applied and measurement temperature was 24 °C. Error bars indicate the standard deviation from five measurements.

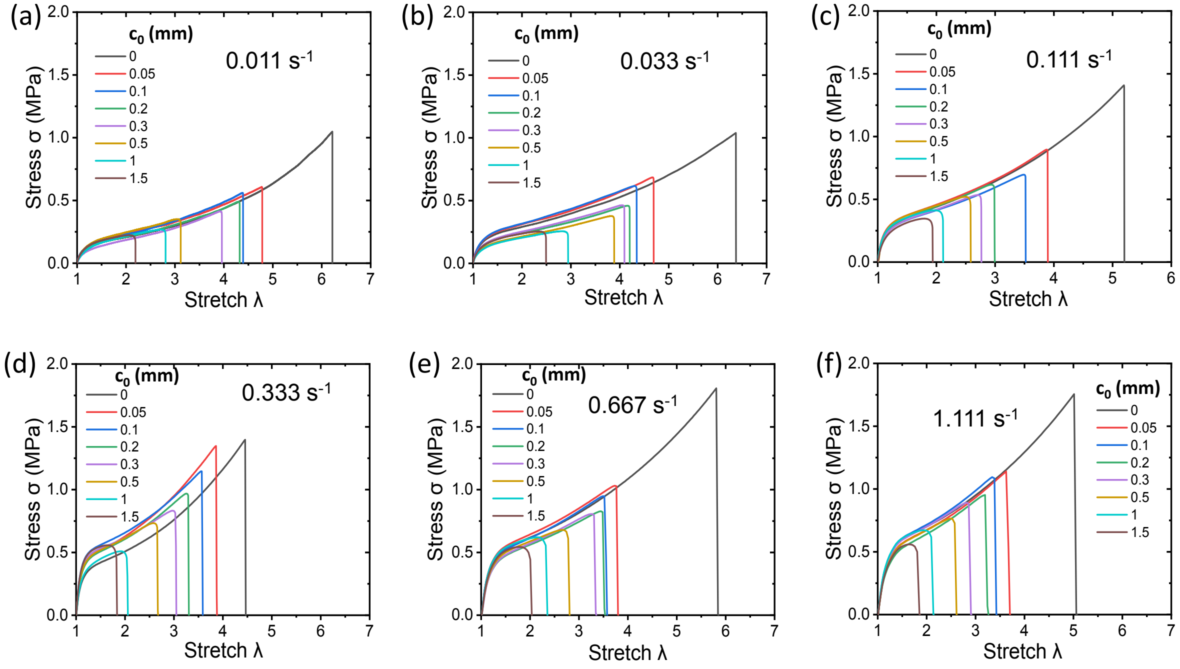

Figure S5. Tensile behavior of P(NaSS-*co*-MPTC)-2.1-0.1 with different initial crack length c_0 at various strain rates: 0.011 s^{-1} (a), 0.033 s^{-1} (b), 0.111 s^{-1} (c), 0.333 s^{-1} (d), 0.667 s^{-1} (e), and 1.111 s^{-1} (f). The measurements were conducted at a temperature of 24 °C.

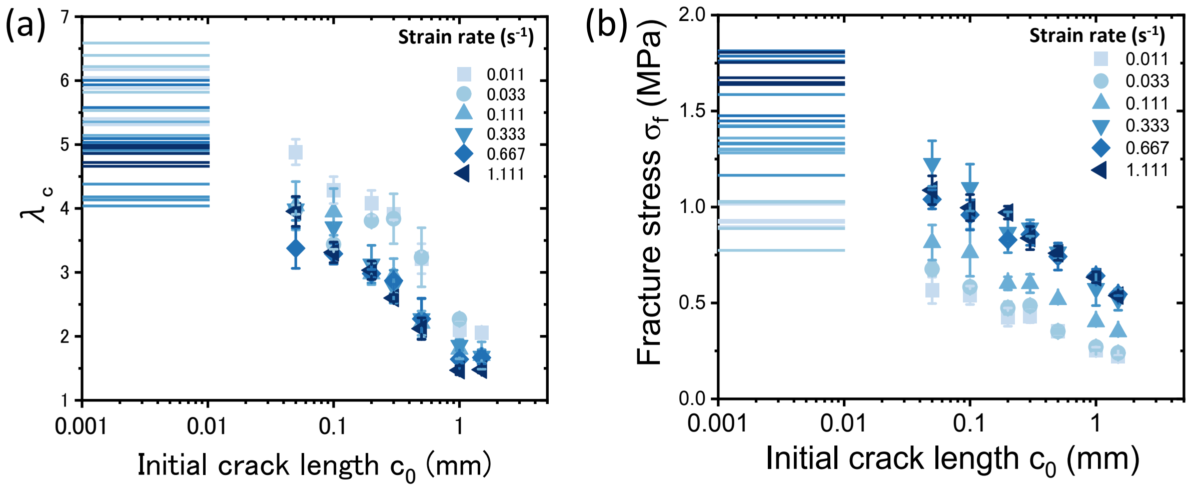

Figure S6. Crack sensitivity of P(NaSS-*co*-MPTC)-2.1-0.1 at various strain rates, estimated by critical stretch ratio of crack propagation (λ_c) and fracture stress (σ_f). (a) λ_c as a function of initial crack length c_0. (b) σ_f as a function of c_0. Horizontal lines represent the values for samples without precuts (five measurements). The error bar is standard deviation from five measurements. The measurement temperature was 24 °C.

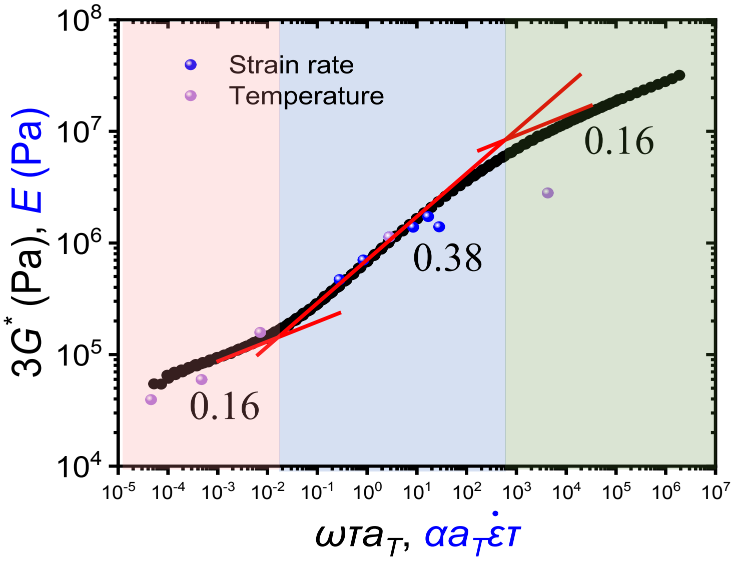

Figure S7. Matching the Young's modulus from tensile tests with the linear rheology master curve (reference temperature: 24 °C) for complex modulus |*G| by shift factors αa_T**. From these curves, we estimated the equivalent strain rates relative to the main relaxation time, $\boldsymbol{\alpha a_T \dot{\varepsilon} \tau}$. The $\alpha = \omega/\dot{\varepsilon}$ is close to 2π for measurements at 24 °C,[40] and a_T represents the temperature-dependent shift factor determined from the linear rheology (Figure 1c). Considering the PA gels are incompressible materials, the relationship $E = 3|G^*|$ was applied.

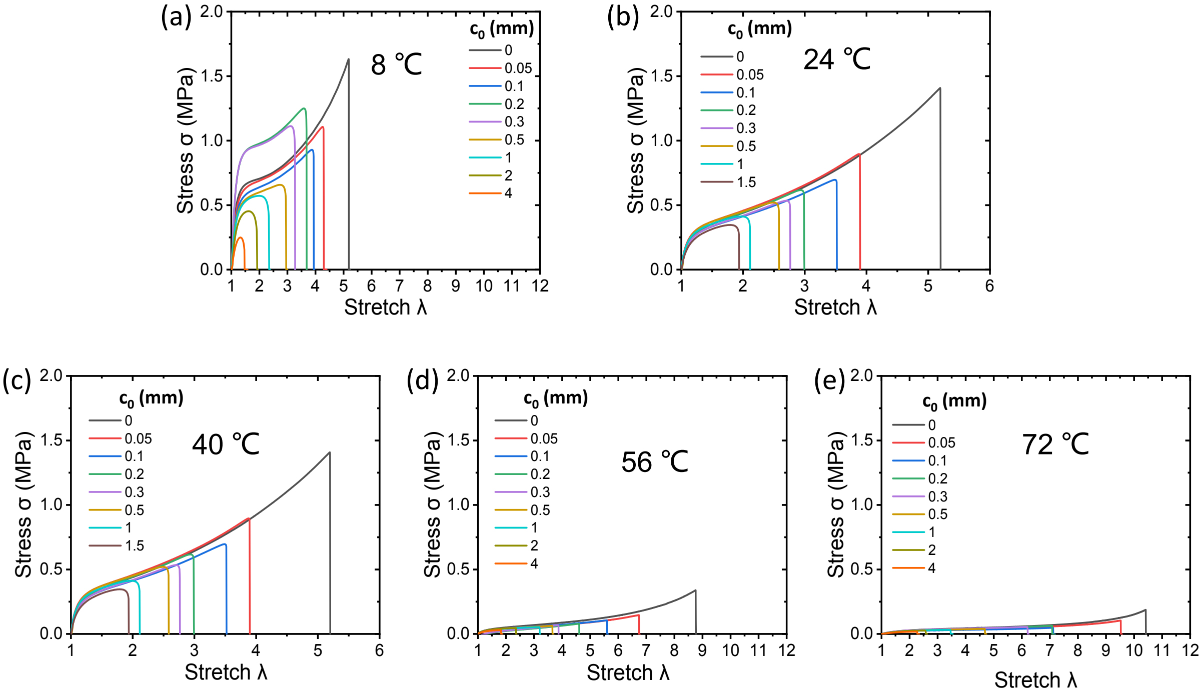

Figure S8. Tensile behavior of P(NaSS-*co*-MPTC)-2.1-0.1 with different initial crack length c_0 at various temperatures: 8 °C (a), 24 °C (b), 40 °C (c), 56 °C (d), and 72 °C (e). A strain rate of 0.111 s^{-1} was applied.

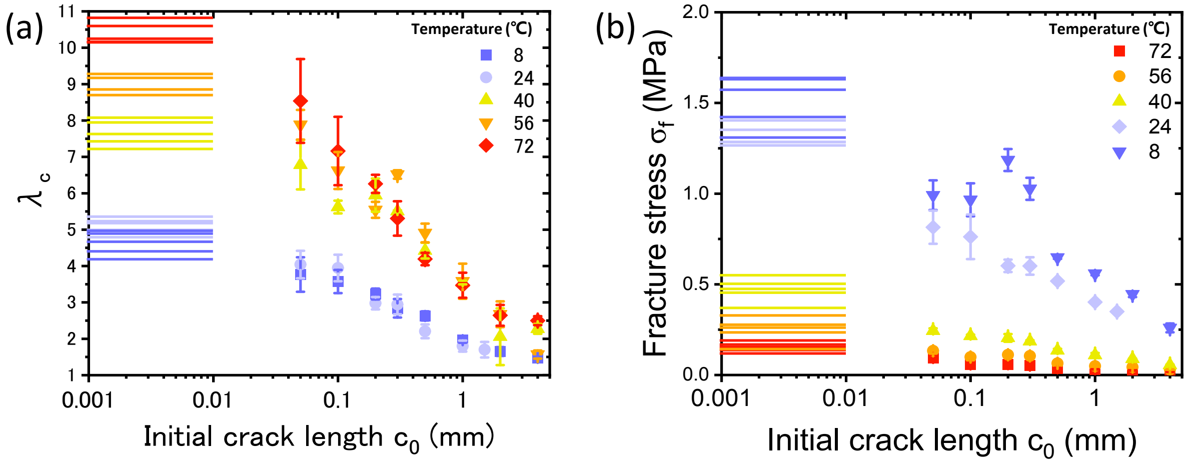

Figure S9. Crack sensitivity of P(NaSS-*co*-MPTC)-2.1-0.1 at various temperatures, estimated by critical stretch ratio of crack propagation (λ_c) and fracture stress (σ_f). (a) λ_c as a function of initial crack length c_0. (b) σ_f as a function of c_0. Horizontal lines represent the values for samples without precuts (five measurements). The error bar is standard deviation from five measurements. A strain rate of 0.111 s^{-1} was applied.

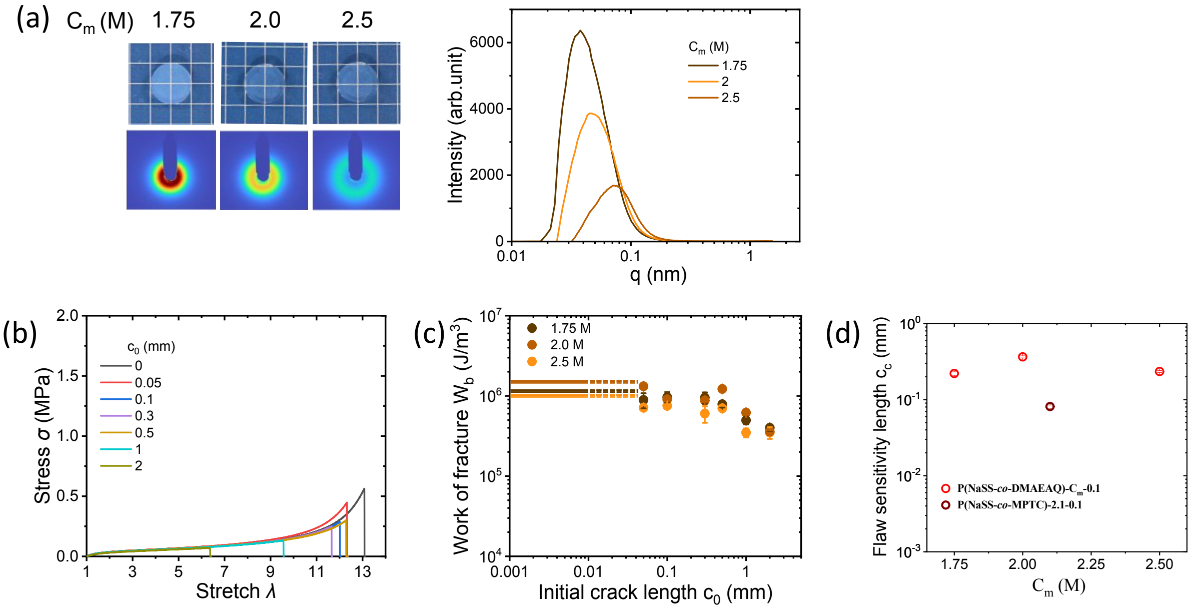

Figure S10. The c_c for P(NaSS-*co*-DMAEA-Q)-C_m-0.1 gels. (a) Appearance and SAXS measurement results of P(NaSS-*co*-DMAEA-Q)-C_m-0.1. (b) Nominal stress-stretch ratio curves of hydrogels with different initial crack lengths (C_m = 2.0 M as an example). A strain rate of 0.111 s^{-1} was applied and measurement temperature was 24 °C. (c) The work of fracture W_b estimated from (b) as a function of initial crack length c_0 for varying C_m. (d) c_c as a function of C_m. The light blue point represents the c_c of P(NaSS-*co*-MPTC)-2.1-0.1.

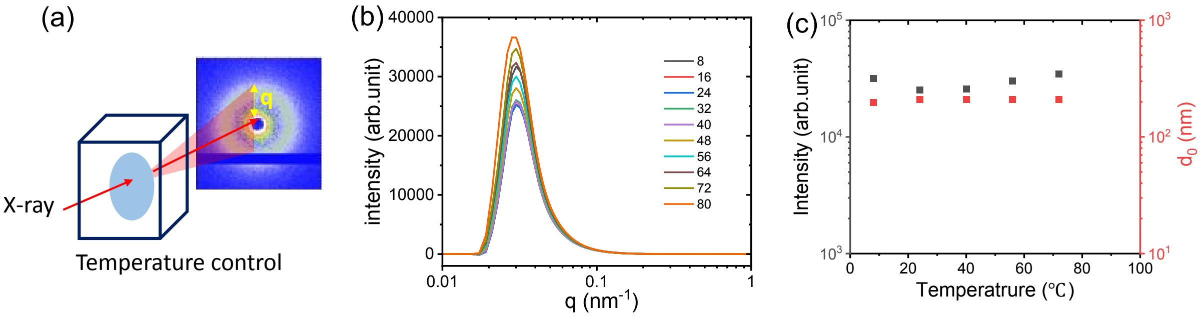

Figure S11. Effect of temperature on phase-separated structure, using P(NaSS-*co*-MPTC)-2.1-0.1 as an example. (a) Schematic diagram of temperature-dependent SAXS measurement. (b) 1D SAXS scattering profiles at varying temperatures. (c) Peak scattering intensity I_{peak} and d-spacing of hard or soft phase domains (d_0) as a function of measurement temperature.

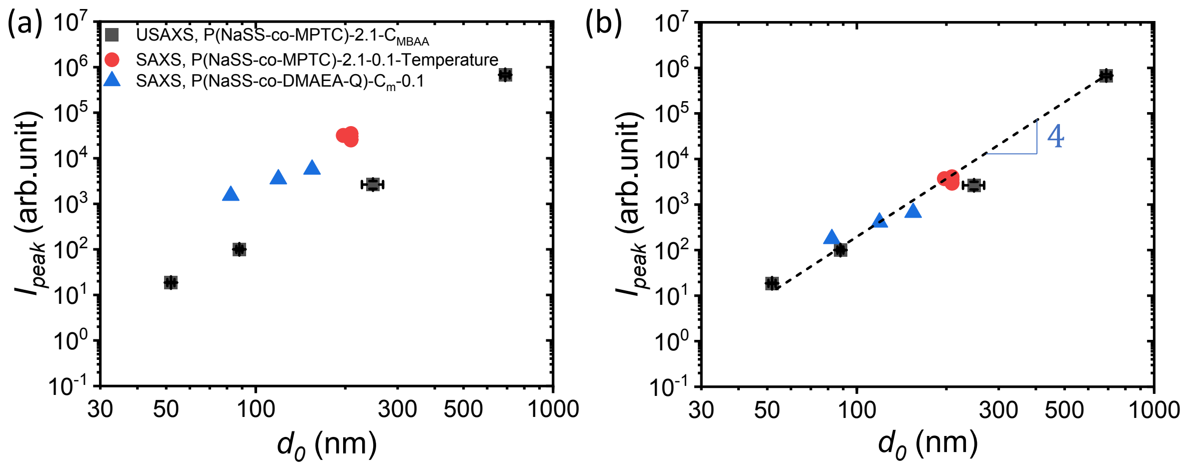

Figure S12. Correlation between SAXS/USAXS peak intensity I_{peak} and d-spacing of hard or soft phase domains (d_0) in water-equilibrated PA gels. These hydrogels were prepared with different chemical cross-linker content (P(NaSS-*co*-MPTC)-2.1-C_{MBAA}), tested under varying tensile temperature (P(NaSS-*co*-MPTC)-2.1-C_{MBAA}-Temperature), and fabricated with different monomer types and monomer concentrations (P(NaSS-*co*-DMAEA-Q)-C_m-0.1). (a) Relationship between I_{peak} and d_0 estimated by USAXS and SAXS measurements. (b) Master curve of I_{peak} against d_0 by vertically shifting the data in (a). Each dataset is represented by the same symbol and color. Error bars indicate the standard deviation from three measurements.

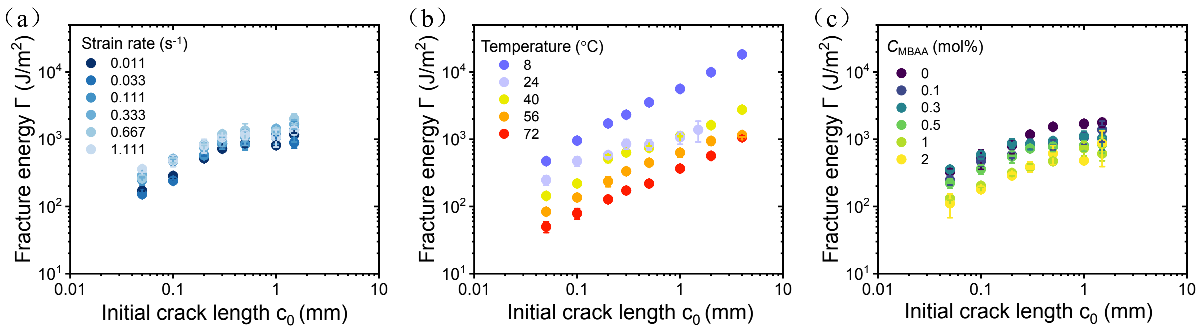

Figure S13. Fracture energy Γ as a function of initial crack length c_0 for the single-edge crack test. (a) P(NaSS-*co*-MPTC)-2.1-0.1 measured at varying strain rates under 24 °C. (b) P(NaSS-*co*-MPTC)-2.1-0.1 measured under varying temperatures at a strain rate of 0.111 s^{-1}. (c) P(NaSS-*co*-MPTC)-2.1-C_{MBAA} measured at a strain rate of 0.111 s^{-1} under 24 °C.

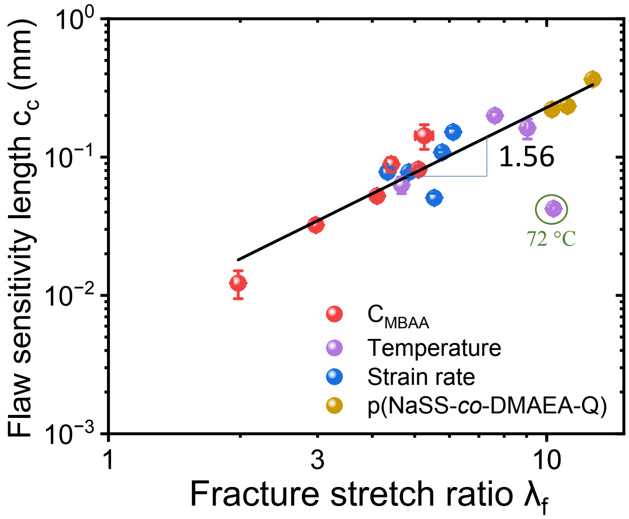

Figure S14. The c_c as a function of fracture stretch ratio of unnotched sample (λ_f). The green-circled point represents P(NaSS-*co*-MPTC)-2.1-0.1 measured at 72 °C. Its deviation from the fitting line may result from chain slippage at high temperatures, as the hydrogel is lightly crosslinked.
